# Piloting a minimum data set for older people living in care homes in England: a developmental study

**DOI:** 10.1093/ageing/afaf001

**Published:** 2025-01-15

**Authors:** Adam L Gordon, Stacey Rand, Elizabeth Crellin, Stephen Allan, Freya Tracey, Kaat De Corte, Therese Lloyd, Richard Brine, Rachael E Carroll, Ann-Marie Towers, Jennifer Kirsty Burton, Gizdem Akdur, Barbara Hanratty, Lucy Webster, Sinead Palmer, Liz Jones, Julienne Meyer, Karen Spilsbury, Anne Killett, Arne T Wolters, Guy Peryer, Claire Goodman

**Affiliations:** Wolfson Institute of Population Health, Queen Mary University of London, London, UK; Academic Centre for Healthy Ageing, Barts Health NHS Trust, London, UK; Personal Social Services Research Unit (PSSRU), University of Kent, Canterbury, Kent, UK; Improvement Analytics Unit, the Health Foundation, London, UK; Personal Social Services Research Unit (PSSRU), University of Kent, Canterbury, Kent, UK; Improvement Analytics Unit, the Health Foundation, London, UK; Improvement Analytics Unit, the Health Foundation, London, UK; Improvement Analytics Unit, the Health Foundation, London, UK; Our Future Health, London, UK; Academic Unit of Injury, Recovery and Inflammation Sciences, School of Medicine, University of Nottingham, Nottingham NG7 2UH, UK; NIHR Applied Research Collaboration-East Midlands (ARC-EM), Nottingham, UK; Health and Social Care Workforce Research Unit, Kings College London, London, UK; School of Cardiovascular and Metabolic Health, College of Medical, Veterinary and Life Sciences, University of Glasgow, Glasgow, UK; Centre for Research in Public Health and Community Care (CRIPACC), University of Hertfordshire, College Lane, Hatfield, UK; Population Health Sciences Institute, Newcastle University, Newcastle upon Tyne, UK; Academic Unit of Injury, Recovery and Inflammation Sciences, School of Medicine, University of Nottingham, Nottingham NG7 2UH, UK; Personal Social Services Research Unit (PSSRU), University of Kent, Canterbury, Kent, UK; National Care Forum, London, UK; School of Health and Psychological Sciences, City University of London, London, UK; School of Healthcare, Faculty of Medicine and Health, University of Leeds, Leeds, UK; NIHR Applied Research Collaboration Yorkshire and Humber (YHARC), Bradford, UK; School of Health Sciences, University of East Anglia, Norwich, Norfolk,UK; Cumbria, Northumberland, Tyne and Wear NHS Foundation Trust, Newcastle upon Tyne, UK; School of Health Sciences, University of East Anglia, Norwich, Norfolk,UK; Centre for Research in Public Health and Community Care (CRIPACC), University of Hertfordshire, College Lane, Hatfield, UK; NIHR Applied Research Collaboration-East of England (ARC-EoE), Cambridge, UK

**Keywords:** care homes, minimum dataset, data linkage, quality of life, digital care record, older people, qualitative research

## Abstract

**Background:**

We developed a prototype minimum data set (MDS) for English care homes, assessing feasibility of extracting data directly from digital care records (DCRs) with linkage to health and social care data.

**Methods:**

Through stakeholder development workshops, literature reviews, surveys and public consultation, we developed an aspirational MDS. We identified ways to extract this from existing sources, including DCRs and routine health and social care datasets. To address gaps, we added validated measures of delirium, cognitive impairment, functional independence and quality of life to DCR software. Following routine health and social care data linkage to DCRs, we compared variables recorded across multiple data sources, using a hierarchical approach to reduce missingness where appropriate. We reported proportions of missingness, mean and standard deviation (SD) or frequencies (%) for all variables.

**Results:**

We recruited 996 residents from 45 care homes in three English Integrated Care Systems. 727 residents had data included in the MDS. Additional data were well completed (<35% missingness at wave 1). Competition for staff time, staff attrition and software-related implementation issues contributed to missing DCR data. Following data linkage and combining variables where appropriate, missingness was reduced (≤4% where applicable).

**Discussion:**

Integration of health and social care is predicated on access to data and interoperability. Despite governance challenges we safely linked care home DCRs to statutory health and social care datasets to create a viable prototype MDS for English care homes. We identified issues around data quality, governance, data plurality and data completion essential to MDS implementation going forward.

## Key Points

- Digital care records (DCRs) and health and social care datasets contain a range of information that can help provide a more complete picture of residents.
- We developed and implemented a minimum dataset linking care home DCRs to statutory health and social care records.
- Information governance for linking data across multiple data owners and data processors is complex and time-consuming.
- Standardisation across DCRs systems would enable data to be used more effectively across the care home sector.
- Establishing shared priorities across key stakeholders interested in care home data is essential to effective minimum data set (MDS) implementation.

## Background

Care homes provide around-the-clock residential care for people whose needs cannot be met by visiting care. Older people living in care homes often have needs defined by one or more frailty, multiple long-term conditions, disability or cognitive impairment [1]. Homes can be registered as with or without nursing depending on whether they employ registered nurses to oversee and provide complex healthcare. In England, there are around 372 000 care home places [2].

Day-to-day care for residents generates abundant data spread across records held by care homes, statutory social care organisations, the National Health Service (NHS), residents and their families [3, 4]. As records become increasingly digitised, there is an opportunity to collate data to inform decisions about commissioning, care planning and delivery, review and funding at the micro (individual resident), meso (care home and regional system) and macro (national system) levels [4].

Care home residents were amongst those most adversely affected by COVID-19, and the sector was devastated by outbreaks [5]. At pandemic outset, England lacked even rudimentary data on how many people lived in care homes to track COVID-19 incidence [6]. Emergency legislation, now repealed, enabled collated datasets and recognition of their potential to inform and transform care.

In other countries, Minimum Data Sets (MDSs) for care homes already exist. The most widely recognised of these are the US Medicare (MDS 3.0) [7] and InterRAI, deployed in multiple jurisdictions [8]. Implementation of MDSs is influenced by mandates and financial incentives supported by ongoing training to motivate staff to engage with MDS completion; the extent to which completion is built into the working practices, monitoring and record systems of all staff (including visiting professionals); and digital recording systems that care home staff use to document and discuss care [9]. At the time of writing, there is no national mandate or incentive framework for implementation of an MDS in any of the four UK nations, although plans are underway to standardise some aspects of social care data collection in England [10].

Against this background, we set out to pilot a prototype MDS for English care homes for older people, focusing on homes currently using digital care records (DCRs) [11]. Our objectives were to (i) assess feasibility of extracting data directly from DCRs and linking these to routinely collected health and social care data to populate a pilot care home MDS, (ii) to assess quality and completeness of MDS data and (iii) describe barriers and facilitators to implementation and use. In this article, we address the first two of these objectives. Implementation and use by care home staff and external stakeholders are addressed in a second paper [12].

## Methods

This was a mixed-methods pilot of a prototype MDS. A full protocol is published elsewhere [11].

### Sampling and resident recruitment

We aimed to recruit 20 care homes for older people in each of three Integrated Care Systems (ICSs), totalling 60 homes. ICSs are regional partnerships between NHS organisations, local government and others, including third sector and social enterprises, which are responsible for co-ordinating and commissioning care in England. From the 42 English ICSs, we chose three—in the South East, East Midlands and North East—to sample different geographies, socio-economic deprivation indices and care configurations. Assuming an occupancy rate of 90%, the sample size required for a true representation of the finite older care home population in each of the ICSs, with 90% confidence and 5% margin of error, was 262–268 residents per ICS [11].

Care homes were eligible for inclusion if using DCRs from one of two participating DCR software companies. Initial approaches were made by email, telephone and in-person, with homes recruited from those responding positively.

All permanent residents of participating care homes were eligible. We excluded residents receiving respite or temporary/short stay care to minimise burden for people undergoing acute transitions and residents identified as in the last few days of life by care home staff to protect residents and families at a difficult time. Consent was obtained from residents to access and extract pseudonymised data from their care home, health and social care records and, separately, to link these. Capacity to provide consent to participate was assessed by a researcher at first meeting. For those without capacity, we asked care home staff to send a letter to a family member or friend who could act as a personal consultee as defined by the Mental Capacity Act. Consultee discussions were conducted either face-to-face or by telephone.

### Selecting items for inclusion in the prototype MDS

MDS development was based upon a review of international research literature summarising outcome measures used in care home studies [13]; a review of measures used in UK care home randomised controlled trials [14]; a systematic review on how contextual factors influence research processes, including data collation in care homes [15]; a series of consultation activities with stakeholders comprising care home managers and staff, and clinical specialists in healthcare of older people and primary care [16–18]; public involvement activity with care home residents, staff and family carers [19]; a survey of data currently collected and collated by English care homes [3]; and a scoping review of published MDSs. From there, we developed nine core principles to govern development and implementation of a care home MDS, previously published [20] (reproduced in Appendix 1).

A corollary of these findings was that a major barrier to implementing an existing MDS already deployed in other jurisdictions, such as interRAI or MDS 3.0, was the need for care homes to stop using existing DCR software and data approaches to start using these products. None of our care home stakeholders were motivated to do so. Additionally, to form a complete dataset, interRAI or MDS 3.0 would either have to duplicate or replace data held in NHS records. The ability to draw from and connect with data already held was seen as important based upon our stakeholders. Therefore, based upon the nine core principles, we compiled an aspirational prototype MDS containing agreed information and a plan for which routine datasets we hoped to collect these from [11] (summarised in Appendix 2). The systematic review of how contextual factors influence research processes [15] informed our approach to MDS implementation. Having established what an aspirational MDS should contain, we then met with DCR providers to explore the variables contained in their datasets.

### Digital care records

19 DCR software providers are, at the time of writing, accredited by NHS England (NHSE) for use in English care homes [21]. We worked with the independent Care Software Providers Association (https://caspa.care) to identify two leading care management software providers, who, between them, provide care software to 9500 of the circa 17 000 care homes in the UK. Through an initial mapping exercise, based on demonstration of a ‘standard’ user interface by the software providers, we identified variables from the aspirational MDS likely to be included in DCRs.

A dummy data extract from both software providers, completed in summer of 2022, identified several variables collected in free text or non-standardised formats. To address gaps in the MDS left by these, which could not be addressed through routine NHS and social care data, additional measures were added to each software system. These included seven validated measures of: ‘delirium’ (I-AGeD) [22]; ‘cognitive impairment’ (MDS Cognitive Performance Scale (MDSCPS)) [23]; ‘functional independence’ (Barthel index) [24]; and ‘quality of life’ (QoL) from the Adult Social Care Outcomes Tool Proxy (ASCOT-Proxy-Resident) [25, 26], EuroQol 5 domain 5 level proxy version (EQ-5D-5L Proxy 2) (EuroQol) [27], ICECAP-O [28] and QUALIDEM [29].

The QoL measures were selected based on evidence of use in care homes, psychometric properties [30], relevance to different QoL constructs (health, social care, dementia and older people) and advice from stakeholder consultations and public involvement activity. Our decision to include QUALIDEM, rather than DEMQoL, as a dementia-specific QoL measure was based on rankings by stakeholders, published in full elsewhere [18]. Taking into account the high prevalence of cognitive impairment in care home residents [1], proxy versions were used. We further included the ASCOT pain item and low mood/anxiety subscale [31], as well as a question to rate overall QoL on a 7-point scale. This overall question was for resident completion where possible or otherwise by staff proxy. The type of help needed by the resident, if any, was recorded.

Researchers provided specifications for the user interface format, data extract and outputs for these measures, which were then implemented by software providers and tested by researchers using a pilot interface, with revisions as needed. In this process, it became evident that some specifications were not possible in both systems due to differences, for example, in how they dealt with missing data and/or because requirements were incompatible with a system’s usual function or output.

Researchers met with care home staff to describe and explain the additional variables and to highlight the need for these to be inputted manually in addition to usual care records. For routinely collected variables, data were extracted from existing records without additional input from care home staff, in the format (s) used by care homes and in an output format feasible for each software provider. This minimised burden on care homes and software providers but meant researchers had to clean raw data and derive variables.

All DCR variables were collected twice, six months apart, in March–June and September–November 2023. We collected a small amount of data directly from care homes through a short online survey at baseline to better understand context of care, including a number of beds, residents, self-funding residents and staff employed by the care home.

### Routinely collected health and social care datasets

We aimed to access the following data sources: general practice electronic medical records and prescribing data, hospital administrative data, operational datasets from emergency services, urgent care and community health, data from local authorities on social care funding, and data from Care Quality Commission (CQC). We expected to access some of these sources at national (e.g. administrative hospital data) and others at local (e.g. community health) level (Appendix 2).

We developed a data flow diagram (Appendix 3) and legal bases for data sharing (Appendix 4).

### Data management and linkage

As the Improvement Analytics Unit based at The Health Foundation (THF) led data management and linkage, data were hosted on THF’s secure ISO27001/DSPT accredited Data Analysis Platform (DAP). Data were stored in AWS S3 buckets, which only Data Managers and approved project data analysts could access. Access to data was controlled by data managers.

For extracts of health and social care information held by different data controllers to be created, pseudonymised and shared with THF, we securely transferred to software providers a unique NHS number salt key to enable pseudonymisation of subjects in the study. A separate salt key was used to pseudonymise the CQC location identifier (unique for each home). Both salt keys used the SHA256 hashing algorithm. Care home pseudonymisation minimised risk of re-identification of individuals based on location. Care home software providers securely transferred extracted DCRs and pseudonymised NHS numbers and care home identifiers for included residents. Data managers isolated pseudonymised NHS numbers and used a pre-computed rainbow table (password cracking tool) of hashed NHS numbers and salt combinations to determine actual NHS numbers of subjects. These were securely transferred to data processors of health and social care data to enable extraction of relevant records of consented residents. Salt keys were separately transferred so data processors could pseudonymise NHS numbers and care home identifiers in extracted health and social care information. Pseudonymised records were securely transferred to THF once all other identifiers were removed.

Non-personal, aggregated care home-level online survey data from care homes in the study were securely transferred to THF by University of Kent and pseudonymised by THF.

The salt keys, rainbow table of hashed NHS number and salt combinations, and data from the survey of care providers with clear CQC location identifiers from University of Kent were stored in a location accessible only by Data Managers, separated from the extracted pseudonymised DCRs and health and social care records, and deleted after the datasets were linked.

Once data were received from data processors, the data was checked and cleaned, and variables were derived (derivation described in Appendix 5a). Data cleaning and variable derivation code is published on Github: https://github.com/HFAnalyticsLab/DACHA. Datasets were linked via pseudonymised NHS numbers and pseudonymised CQC location identifiers.

### Stakeholder engagement

We engaged technical experts within NHSE and the ICSs on information governance, data access and availability. We also engaged with wider stakeholders within each ICS to gain support for the project, facilitate data sharing and inform analyses to be conducted on the MDS. Stakeholders included care home managers, staff, residents and family members, GPs and local decision-makers within the NHS and local authorities. We also engaged with DHSC and NHSE programme teams (Enhanced Health in Care Homes and Ageing Well) at a national level to understand how an MDS could inform national policy priorities.

Importantly, initial buy-in from the three ICSs at the start of the study, three years before resident consent and data collation began, dissipated by the time discussions around data access started. This was due both to key stakeholders leaving and competing priorities for limited analytical and IG resources. Stakeholders who were able to influence data access and had clinical contact with care homes to inform discussions about data analysis differed between ICSs.

### Deriving MDS variables

We designed a person-level, one-row per-resident MDS. The date on which additional care home measures were first completed by care home staff, or 1 June if missing, was the index date for all other MDS variables. The Elixhauser list of comorbidities [32, 33] and a validated list of frailty syndromes [34] were identified from hospital admission data using ICD-10 codes for 3 years prior to each resident’s index date. Potentially avoidable admissions were those due to a list of conditions originally developed by the CQC [35].

Healthcare utilisation was collated for the year before the index date. By exception, ambulance activity was only calculated for the period between the first and second MDS measurements. ‘Out of hours’ was defined as 18:00–08:00 and ‘long attendance’ as being at Emergency Departments for 12 hours or longer. All variable derivations are detailed in the final MDS data specification (Appendix 5).

### Data analysis

Where variables were available from multiple data sources, we compared levels of completeness and agreement. To determine which data source(s) would populate the final MDS, we constructed a hierarchy based on data quality and expert opinion. We distinguished between variables with a universal definition across datasets, such as date of birth or sex, and those which could be defined in multiple ways or vary over time, such as cognitive impairment or delirium. For the first category, we created a hierarchy collapsing all sources into one final variable. For the second, we presented a comparison but retained all variables in the final MDS. By exception, we took an additive approach to dementia. We used Personal Demographic Service (PDS) [36] as the master index based on NHSE guidance and Secondary Uses Service (SUS) [37] where data were unavailable in PDS. The exception was ethnicity, where we used the care home record in the first instance, as self-reported ethnicity is more accurate than observational data commonly found in secondary care records [38].

Date of death can often generate disagreement between systems, mainly because dates of death notification and certification by the Office of National Statistics may differ [39] . However, they rarely vary >30 days, with negligible effect on analysis.

To understand the information contained within the MDS, we reported proportions of missingness, mean and standard deviation (SD) or frequency (%) as appropriate. We also derived two-way tables to provide worked examples of opportunities for more detailed descriptive statistics from the MDS, focusing on emergency attendance and ambulance activity based on discussions with stakeholders described above.

Evaluation of psychometric properties of the QoL measures (ASCOT-Proxy-Resident, ICECAP-O, EQ-5D-5L Proxy 2, QUALIDEM) are reported elsewhere [40–42]. These analyses identified limitations around using QUALIDEM in an older adult care home MDS, so we do not report QUALIDEM results here.

The analysis code is published on Github: https://github.com/HFAnalyticsLab/DACHA. We used R version 4.0.2, SAS Enterprise Guide version 8.3 (NHS and social care routine data), and Stata version 18 (DCR data).

## Results

We recruited 996 residents from 45 care homes (Table 1). Working from lists of care home providers using particular DCR software meant brokering relationships with care homes often new to research. Success was greatest in ICS Area 1 because of long-established relationships between the researchers and their local care home community.

**Table 1:** Actual versus target recruitment by ICS area.

ICS Area	Target recruitment	Actual recruitment
1	20 care homes	19 care homes
	320 residents	537 residents
2	20 care homes	15 care homes
	358 residents	286 residents
3	20 care homes	11 care homes
	292 residents	173 residents
Total	60 care homes	45 care homes
	970 residents	996 residents

**Figure 1:**
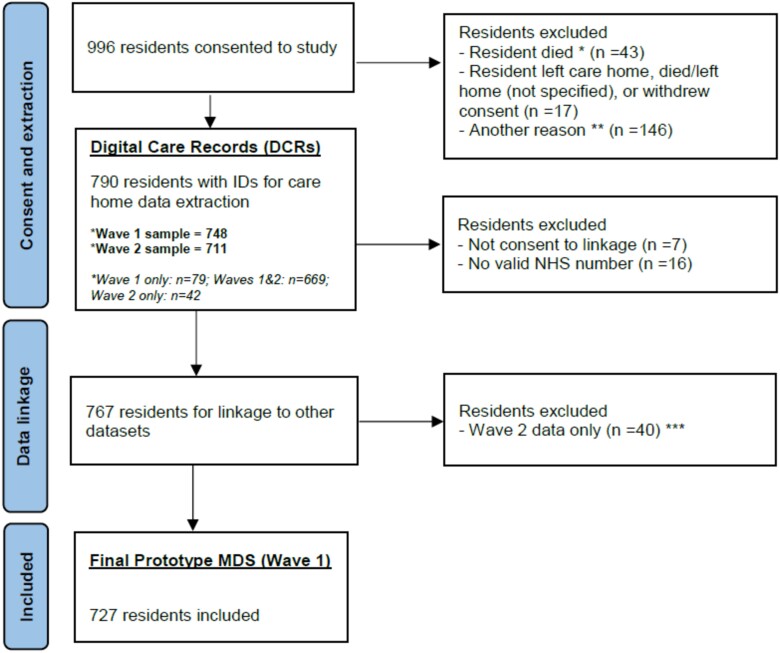
Flow diagram of residents from recruitment into final prototype MDS (Wave 1).

From 996 eligible residents, 767 had data extracted which could be linked. Of these, 727 residents had complete data for baseline DCR data collection and were included in the final prototype MDS (Figure 1). Of these, 696 had a DCR with a valid CQC identifier enabling linkage to care home-level data from CQC records and the online survey.

### Digital care records from care homes

First, we describe DCR data extracted from care homes (Table 2) before we consider accessed datasets and subsequent linkage into the final prototype MDS. Table 2 includes data for the 790 residents (see Figure 1, under consent and extraction) who provided consent and had a valid ID for data extraction (*n* = 748 at Wave 1, *n* = 711 at Wave 2). For residents with complete data at Wave 1 but not Wave 2, most were attributable to resident death or care home drop-out from the study between waves (see Appendix 6).

**Table 2:** Overview of data extracted from DCRs.

			Wave 1			Wave 2			
MDS	Variable	Categories (if applicable)	*n*	Mean, SD (range) or freq. %	% Missing data^a^	*n*	Mean, SD (range) or freq. %	% Missing data^b^	Other comments
1	Ethnicity	Asian or Asian British White or White British	163	≤4% ≥96%	78.2%	122	≤4% ≥96%	82.8%	Derived variable. Coded differently by the two software providers.
1	Religion	No religion Christian Buddhist Other	119	11.8% ≥76.5% ≤5% 6.7%	84.1%	89	9.0% ≥79% ≤6% ≤6%	87.5%	Derived variable. Coded differently by the two software providers.
1	Marital Status	Married/cohabit Widowed Divorced/single/separated	114	35.1% 50.9% 14.0%	84.8%	120	34.2% 49.2% 16.6%	83.1%	Not available for Provider 2 (100% missing).
1	First Language	English Other	170	≥96% ≤4%	77.3%	134	≥96% ≤4%	81.2%	Not available for Provider 2 (100% missing).
1	Deprivation of Liberty	No Yes	689	81.3% 18.7%	7.9%	560	76.8% 23.2%	21.2%	
1	Weight	20-35 kg 36-50 kg 51-65 kg 66-80 kg 81-95 kg 96-110 kg 111-125 kg 126-140 kg	586	1.2% 19.8% ≥39.4% 26.3% 7.7% 3.6% ≤1% ≤1%	21.7%	573	1.9% 19.2% ≥37.6% 26.9% 9.1% 3.3% ≤1% ≤1%	19.4%	Derived variable. Provider 1: numerical, Provider 2: categorical. Majority of missing data (≥95%) are from Provider 2.^c^
1	Height	111-125 cm 126-150 cm 151-170 cm 171-190 cm 191-210 cm	738	1.1% 9.8% ≥70.1% 18.0% ≤1%	1.3%	674	1.2% 11.0% ≥68.2% 18.6% ≤1%	5.2%	Derived variable. Provider 1: numerical, Provider 2: categorical.
1	DNACPR status	No Yes	745	21.1% 78.9%	0.4%	707	18.7% 81.3%	0.6%	
1	Power of attorney	No Yes	170	62.4% 37.6%	77.3%	168	60.7% 39.3%	76.4%	Not available for Provider 2 (100% missing).
3	Length of stay, days		747	873.7, 807.5 (<50 to >8000)	0.1%	710	1011.4, 815.6 (<50 to >8000)	0.1%	Derived from date of entry to home. For Provider 2, only have month/year, so set to the 1st of the month. Data quality was improved across waves, since able to verify and correct anomalous dates (e.g. all set to the same date within a care home). Nevertheless, one case (*n* = 1) omitted due to likely data entry error.
4	Textured food/diet	IDDSI 7 – regular IDDSI 7 – easy to chew IDDSI 6 – soft & bite sized IDDSI 5 – minced & moist IDDSI 4 – pureed IDDSI 3—liquidised	687	≥71.3% 5.1% 9.5% 7.1% 6.0% ≤1%	8.2%	611	≥68.3 6.7% 8.8% 8.2% 7.0% ≤1%	14.1%	
4	Textured drink/fluid	IDDSI 0 – thin IDDSI 1 – slightly thickened IDDSI 2 – mildly IDDSI 3 – moderately IDDSI 4 – extremely	672	≥89.9 4.5% 3.6% ≤1% ≤1%	10.2%	605	89.2% 4.8% 5.0% ≤1% None	14.9%	
4	Cognitive impairment	Very severe Severe Moderately severe Moderate Mild Borderline intact Intact	630	12.5% 14.4% 12.9% 17.9% 14.0% 9.4% 18.9%	15.8%	479	14.6% 17.3% 12.9% 18.6% 12.1% 7.7% 16.7%	32.6%	Added to software for the MDS pilot. The MDS CPS is calculated from five items: comatose, problem with short-term memory, cognitive skills for daily decision making, being understood by others and eating ADL. Scored per Morris et al. [43].
4	Waterlow Score		542	17.8, 7.3 (5 to 44)	27.5%	473	18.3, 7.3 (5 to 43)	33.4%	Score of ≥10 indicates risk for pressure ulcer, with high risk ≥15 and very high risk ≥20. Full score range from 0 to 64.^d^
4	Braden Score		345	16.6, 4.0 (7 to 23)	53.9%	277	16.4, 4.0 (8 to 23)	61.0%	Full score range of 6 to 23, with higher scores indicating lower risk of pressure ulcers. Scores ≤10–12 indicate high risk and ≤9 very high risk.
4	Barthel Index		582	41.5, 30.2 (0 to 100)	22.2%	288	34.9, 28.7 (0 to 100)	59.5%	Added to software for the pilot. Score from lowest (0) to highest (2) level of functional independence for each item. These are summed, x5, to create a score from lowest (0) to highest (100) independence.
4	Delirium/I-AGED		601	1.1, 1.8 (0 to 10)	19.7%	466	1.4, 2.1 (0 to 10)	34.5%	Added to software for the pilot. Each of the ten items is scored no (0) or yes (1) and summed to create a scale from 0 to 10. Score of ≥4 indicates delirium [44].
5	ASCOT proxy-resident		503	.83, 0.19 (−.17 to 1)	32.8%	384	.81, 0.19 (−.17 to 1)	46.0%	Added to software for the pilot. Required some recoding to combine. Applied preference weights for ASCOT SCT4 to generate index score from −.17 to 1.0 [45].
5	ASCOT: anxiety and low mood		503	4.0, 1.5 (0 to 6)	32.8%	403	3.9, 1.5 (0 to 6)	43.3%	Added to software for the pilot. Required some recoding to combine.
5	ASCOT: pain		568	2.2, 0.8 (0 to 3)	24.1%	423	2.1, 0.9 (0 to 3)	40.5%	Added to software for the pilot. Required some recoding to combine.
5	ICECAP-O		583	.73, 0.21 (0 to 1)	22.1%	300	.71, 0.22 (0 to 1)	57.8%	Added to software for the pilot. Required some recoding to combine. Provider 2: Data not provided for some residents. Score (0 to 1) calculated using UK index values [46].
5	EQ-5D-5L Proxy 2		650	.33, 0.35 (−.59 to 1)	13.1%	494	.29, 0.34 (−.35 to 1)	30.5%	Added to software for the pilot. Required some recoding to combine. Score calculated using the mapping function to convert to EQ-5D-3L and applied UK index values. The UK value set for the EQ-5D-5L is still being developed [47, 48]
5	ASCS QoL	So good Very good Good Alright Bad Very bad So bad	613	3.1% 26.3% 33.9% 28.9% 5.2% 1.5% 1.1%	18.1%	461	3.9% 25.8% ≥36.1% 27.3% 4.6% 1.3% ≤1%	35.2%	Added to software for the pilot. Required some recoding to combine.
6	NEWS2/ RESTORE2		0	N/A	100%	0	N/A	100%	Provider 1. Included in the software for data capture but no data entered by care homes (100% missing). Provider 2. Not available (100% missing).
6	MUST		169	.8, 1.2 (0 to 5)	77.4%	161	1.0, 1.3 (0 to 4)	77.4%	MUST scored in last 6 months. A MUST score of 1 indicates medium risk and ≥ 2 indicated high risk of malnutrition. Not available for Provider 2 (100% missing).

^a^Wave 1: *n* = 748 (Provider 1 *n* = 170; Provider 2 *n* = 578)

^b^Wave 2: *n* = 711 (Provider 1 *n* = 168; Provider 2 *n* = 543).

^c^Included a flag to indicate cases with multiple entries (*n* = 8) for Provider 2. As date of entry was unavailable, one entry was randomly selected.

^d^Included a flag to indicate cases with multiple entries for an individual (*n* = 69) for Provider 2. As date of entry was not available, one entry was randomly selected.

Where data were already included in routine DCRs, some variables were more complete than others. CPR status was 99.6% complete. Care homes using Software Provider 2’s system did not routinely complete fields including marital status, first language, power of attorney and malnutrition universal screening tool (MUST), which contributed to high levels of missing data. For National Emergency Warning Score 2 (NEWS2) variables, no data were entered by care homes using either software.

The measures added to DCRs for the pilot were more consistently completed compared to those routinely recorded (Wave 1: <35% missing data). This is perhaps to be expected since we required software providers to include these measures across participating homes, whereas homes could choose what routine data to record. We also devoted researcher time to explaining the new variables and the rationale for their inclusion in care home staff.

In comparing Wave 1 and Wave 2, missing data increased by >8% for deprivation of liberty (7.9%–21.2% for waves 1 and 2, respectively). For variables added to DCRs, missing data increased between 10% and 18% from Wave 1 to 2, except for the Barthel Index (increased by 36%) and ICECAP-O (increased by 37%). For Barthel, this was likely due to Provider 2 using a similar, but slightly different, version as their system default, which care homes reverted to using rather than the standardised version added for the study. Provider 2 did not return ICECAP-O data for five care homes at Wave 2.

Even with relatively high completion for QoL measures, there were issues with data quality in Wave 2. Provider 2 ‘carried over’ Wave 1 scores; therefore, care homes had to manually overwrite prepopulated scores. By contrast, Provider 1 required data entry of new scores for Wave 2. As a result, all but one care home using Provider 2 software had a maximum of two residents with any change in ASCOT-Proxy-Resident score between Wave 1 and 2, whereas only three residents had the same ASCOT Proxy-Resident score across waves for homes using Provider 1’s software.

### Accessed routinely collected health and social care datasets

We were able to retrieve and link data from PDS; SUS Admitted Patient Care, Outpatient and Emergency Care datasets and CQC care home data and supplement this with data from our online survey of care homes as planned. We were additionally able to collect data from the newly available national ambulance [49], adult social care client level [50], and community services (CSDS) [51] datasets. A care home residency table created by Arden & GEM Commissioning Support Unit (CSU) [52] based on PDS data and estimated care home residency dates, and ONS Index of Multiple Deprivation data were also accessed.

Due to information governance constraints, a new data-sharing agreement with NHSE was required, which was signed in October 2023. This delayed access to NHSE datasets and restricted the analysis possible in the remaining time. This also adversely impacted the set-up of data sharing with ICSs.

These datasets were accessed only for consented residents and not for all care home residents in the ICSs as originally planned [11]. In addition to IG challenges, this was primarily because the underlying flow of data previously used to identify care home residents had been replaced, resulting in the complex algorithm [47] for care home identification needing to be redeveloped and validated by NHSE.

We were unable to access GP records because we couldn’t establish data sharing agreements for two of the ICSs in time for the study. In the remaining ICS, we were able to secure some data-sharing agreements with GP practices by working through a CSU, a regional body providing data support to NHS organisations. However, patient data are held by individual GP practices, and we had to liaise with multiple Data Protection Officers within the same ICS. Ultimately, the number of resident records available from GP practices that signed agreements in time was too low to ensure residents could not be re-identified, and therefore, it was not possible to proceed to extraction under General Data Protection Regulations (GDPR). A list of data items we would have accessed from one ICS where we established data sharing agreements, had we been able, is available in Appendix 7.

The inability to collect GP data was a major contributor to the differences between the aspirational and final prototype MDS, summarised in Appendix 8. Other contributors were poor feasibility of extraction from DCRs and high levels of missing data for some items in routine datasets, rendering reliable counts of activity linked to particular conditions or events impossible.

### Creating derived variables in prototype MDS

Due to the absence of GP data, comorbidities were derived from SUS data, using a 3-year lookback period from the index date. We couldn’t derive these for 144 residents (20%) who didn’t have a hospital admission in that period. Activity summaries were reported for the year leading up to the index date, independent of whether residents joined their current care home within this time period. On average, residents in Wave 1 had been living in the current care home for 28.7 months, with 29% having moved in within the year leading up to their index date.

## Hierarchy process

Table 3 presents the variables included in the hierarchy. For universally defined variables, there were high levels of consistency where recorded. Levels of completeness varied widely—from 1% missing for sex in CSDS to 80% missing for ethnicity in the care home record. Overall, the process of using information from several sources to populate the final variable included in the MDS greatly reduced the level of missing data (missingness ≤4% across variables).

**Table 3:** Comparison of variables across data sources to determine hierarchy.

Variable	Category	PDS		SUS		CSDS		Care home residency		Care home record		*n* with non-missing values in both/all relevant data sets and % agreement																		Final^d^	
												SUS vs CSDS		SUS vs PDS		SUS vs CHR		SUS vs care home record		CSDS vs PDS		CSDS vs care home record		PDS vs CHR		PDS vs care home record		All			
		*n*	%	*n*	%	*n*	%	*n*	%	*n*	%	*n*	%	*n*	%	*n*	%	*n*	%	*n*	%	*n*	%	*n*	%	*n*	%	*n*	%	*n*	%
Ethnicity	White	NA		586	81%	570	78%	NA		≥151	≥20%	473	98%	NA		NA		125	99%	NA		92	98%	NA		NA		78	99%	692	95%
	Black or Black British			0	0%	≤5	≤1%			0	NA																			≤5	NA
	Asian or Asian British			0	0%	0	0%			≤5	≤1%																			≤5	NA
	Mixed			≤5	NA	0	0%			0	NA																			≤5	NA
	Other			≤5	NA	≤5	≤1%			0	NA																			≤5	NA
	Missing			135	19%	149	20%			573	79%																			25	4%
Sexa	Female	453	62%	487	67%	504	69%	NA		178	23%	684	100%	NA		NA		240	99%	NA		245	99%	NA		NA		198	99%	542	71%
	Male	175	24%	205	28%	214	29%			73	10%																			225	29%
	Missing	99	14%	35	5%	9	1%			476	65%																			0	0%
Date of birth	Available	628	86%	692	95%	NA		NA		NA		NA		598	100%	NA		NA		NA		NA		NA		NA		NA		≥760	≥99%
	Missing	99	14%	35	5%																									≤5	≤1%
Record of deathb	Present	≤5	NA	9	1%	NA		57	7%	NA		NA		≤5	100%	8	100%	NA		NA		NA		≤5	100%	NA		≤5	100%	58	8%
	Not present	≥720	≥99%	718	99%			670	92%																					709	92%
Dementiac	Yes	NA		394	54%	NA		NA		376	52%	NA		NA		NA		342	75%	NA		NA		NA		NA		NA		514	71%
	No			189	26%					199	27%																			191	26%
	Missing			144	20%					152	21%																			22	3%

^a^Source data sets refer to gender but data are recorded as 0/1 and labelled as male/ female so we understand this to be sex.

^b^Date of death was determined as agreed where the two dates were within 30 days of each other.

^c^Two code lists were used to identify dementia in SUS diagnosis codes (Charlson and frailty; Appendix 9). Additive approach taken where a record with either was identified as dementia present in SUS record.

^d^Collapsed variables were formed using the hierarchy PDS > SUS > CSDS > care home residency > DCR, with exception of ethnicity. For dementia, a record in either SUS or DCR resulted in a record in the final variable.

### Final prototype MDS

Key variables from the final prototype MDS are summarised in Table 4. Appendix 10 shows the full version, which includes two approaches to healthcare utilisation—mean activity across all residents and proportion of residents with at least one event. Appendix 11 contains worked examples, based upon our work with stakeholders, of how data from the MDS could be used to help understand Emergency Department and Ambulance contacts.

**Table 4:** Selected variables from final prototype MDS. Numbers are reported for 727 residents unless otherwise specified.

Domain	Variable	Categories (if categorical)	*n*	Mean (SD) or %
Demographics/characteristics	Ethnicity (final)^a^	White	692	95%
		Black or Black British	≤5	NA
		Asian or Asian British	≤5	NA
		Mixed	≤5	NA
		Other	≤5	NA
		Missing	25	3%
	Sex (final)^a^	Female	513	71%
		Male	214	29%
	Date of birth record (final)^a^	Available	≥720	99%
		Missing	≤5	NA
	Date of death present in record (final)^a^	Present	58	8%
		Not present	669	92%
Palliative care needs	Discussed preferred death location indicator	Yes	18	3%
		No	383	53%
		Missing	326	45%
	Preferred death location	Care home	7	1%
		Care home services with nursing	27	4%
		Care home services without nursing	51	7%
		Hospice	≤5	NA
		Hospital	≤5	NA
		Patient’s own home	16	2%
		Other (not listed)	≤5	NA
		Missing	623	86%
Care home stay	Client funding status	Health funded	7	1%
		Social care funded	18	2%
		Client funded	19	3%
		Joint client and social care funded	96	13%
		Other	≤5	NA
		Unknown in record	77	11%
		Missing	≥505	70%
Residents needs	Cognitive impairment	Borderline intact	56	8%
		Intact	116	16%
		Mild impairment	85	12%
		Moderate impairment	111	15%
		Moderately severe impairment	80	11%
		Severe impairment	88	12%
		Very severe impairment	76	10%
		Missing	115	16%
	Functional independence (Barthel index) (reported for 566 residents/ 22% missing)			41.40 (30.26)
QoL	Ascot Proxy-Resident (reported for 488 residents/ 33% missing)			0.83 (0.19)
	ICECAP-O (reported for 569 residents/ 22% missing)			0.73 (0.21)
	EQ-5D-5L Proxy 2			0.33 (0.35)
Diagnoses (based on previous 3 years hospital admission diagnosis codes) (reported for 583 residents/ 20% missing) apart from ‘dementia (final)’	Dementia (final)^a^		514	71%
	‘Elixhauser conditions’^b^			
	Number of Elixhauser conditions			3.59 (2.34)
	two or more Elixhauser conditions		470	81%
	Anaemia		83	14%
	Congestive heart failure		86	15%
	Chronic pulmonary disease		110	19%
	Depression		129	22%
	Diabetes (complicated and uncomplicated)		127	22%
	Fluid and electrolyte disorders		226	39%
	Hypertension (complicated and uncomplicated)		353	61%
	Hypothyroidism		75	13%
	Liver disease		30	5%
	Obesity		39	7%
	Other neurological disorders		154	26%
	Peripheral vascular disease		47	8%
	Rheumatoid arthritis/collagen vascular diseases		179	31%
	Renal failure		39	7%
	Valvular disease		67	11%
	Weight loss		19	3%
	‘Frailty syndromes’^c^			
	Number of frailty syndromes			2.17 (1.81)
	Cognitive impairment (delirium, dementia, senility)		457	78%
	Anxiety/Depression		168	29%
	Functional dependence		102	17%
	Falls/Fractures		291	50%
	Incontinence		105	18%
	Mobility problems		217	37%
	Pressure ulcers		62	11%
Healthcare utilisation			*n* (people with at least one event)	% who had at least one event
	Elective admissions (1 year history)		65	9%
	Emergency admissions (1 year history)		284	39%
	Potentially avoidable emergency admissions (1 year history)^d^		119	16%
	Emergency department attendances (1 year history)		370	51%
	Community services appointments (1 year history)		608	84%
	Face to face community services appointments (1 year history)		444	61%
	District nursing appointments (1 year history)		398	55%
	Ambulance call outs (1 June—31 October 2023)		197	27%
	Ambulance attendances (1 June—31 October 2023)		195	27%
	Ambulance conveyances (1 June—31 October 2023)		147	20%
Care home characteristic and workforce characteristics	Service type	Nursing	403	55%
		Nursing and Residential	49	7%
		Residential	262	36%
		Missing	13	2%
	Registered bed capacity	<50	211	29%
		50 or more	485	67%
		Missing	31	4%
	CQC rating	Outstanding	72	10%
		Good	511	70%
		Requires improvement	113	16%
		Missing	31	4%
	Years of service registration	<10 years	238	33%
		>10 years	458	63%
		Missing	31	4%

^a^reporting variable as created in the hierarchy process, see Table 3.

^b^Elixhauser list of comorbidities [32,33].

^c^Frailty Syndromes [34].

^d^Potentially avoidable emergency admissions [35].

## Discussion

In the face of substantial challenges, many of which were not unique to this study [53, 54], we accessed information from care home DCRs and safely linked data from multiple sources and data owners to create a viable prototype MDS for English care homes. Our prototype MDS was cross-sectional. Real-world deployment would be longitudinal, with data extracted at regular intervals, balancing the requirements of those funding, planning and delivering services against burden of data completion and collation.

We set out to collate routine administrative health and social care data for all care home residents in participating ICSs, with linkage to DCRs taking place only for those giving consent. This should have been technically feasible using methods outlined in this paper alongside a published algorithm to identify care home residents in routine data [55]. However, the algorithm was under redevelopment at the time of our pilot and couldn’t be validated in time to be incorporated into our data flow. Our final prototype MDS was therefore limited only to residents providing consent to linkage. This may have introduced systematic bias, and data presented here should not be seen as representative of the wider UK care home population. For example, our data on healthcare resource use should be interpreted with caution—we do not know how health status influenced ability to provide consent.

Our data on health status, meanwhile, are limited by lack of access to GP records. This is reflected in lower reported prevalence of common conditions, such as dementia, than in previously published studies, although the prevalence based upon MDS CPS corresponds better to the prevalence cited elsewhere [1, 56]. Long-term conditions such as incontinence and hearing loss, central to understanding healthcare needs in care home residents, are under-recorded in secondary care records [56]. If the MDS presented here is to be of use in practice, incorporating GP data is essential. The challenges encountered in accessing GP data related to information governance and our role as researchers external to the ICS, coupled with time constraints. It was not due to resistance to the principle of data linkage. GP practices work as independent contractors commissioned by the NHS; each practice acts as a data controller for their own patients’ data, and there is, as of yet, no national GP dataset.

Our design repurposed routinely collected care home data to minimise care staff burden and focus on capturing what was important to staff and residents. Where data were central to routine care delivery—such as CPR or Deprivation of Liberty status—they were largely complete. Variables that were incomplete were either regarded as superfluous because care staff know these for their residents (e.g. ethnicity or marital status), captured in free text and difficult to analyse, or difficult to record in a dependent population (e.g. weight). Variables added via external mandate (e.g. NEWS2, included at the request of healthcare providers) [57] were not completed. For variables added to DCRs by our research team for the pilot, we saw initial high completion rates fall during the second wave of data collection. This was multifactorial, with competition for staff time, staff attrition and implementation issues, including a duplicate Barthel Index in some care homes’ software, all contributing. We did not collect data on the amount of staff time spent completing additional variables—this limits our understanding of the quantitative impact of doing so upon their workload. These findings align with previous research on the importance of understanding the context of data collection when working with and interpreting data from social care [4, 58].

An alternative approach to the one used here would be to implement an ‘off the peg’ internationally validated MDS, such as interRAI or MDS 3.0. These would have a number of advantages, including deploying well-established and validated variables, deployed in a consistent way through licenced software, and which are regularly updated through reference to the evolving gerontological literature [8]. It is important, though, to note that this approach would not necessarily be a viable alternative for the UK. It superimposes a new system of data capture onto care homes, not linked to health and social care data held elsewhere and favours health data over QoL and social care data. The issues we addressed around GDPR, the labour-intensive and manual nature of linkage between care home and NHS data, the complex hierarchy of statutory databases into which an MDS has to interdigitate, and the need to train and invest in care home staff over time, would be the same. The interRAI is able to be deployed across multiple care settings, including acute hospitals and domiciliary care [8]—approaches to care records in these sectors in the UK are at least as fragmented as in the care home sector and could benefit from harmonisation, but the complexity of deploying a universal data solution increases with the number of care sectors involved. Previous attempts to use such MDSs in UK research studies found low completion rates, and crucially, a higher burden associated with staff completing them on top of existing data requirements [59, 60]. The work of implementation for uptake and sustained use is as significant and arguably more resource-intensive than we found for our prototype dataset [61]. Dwelling excessively upon such approaches also misses the substantial progress made across health and social care data integration in multiple parts of the UK [62, 63]. The challenge is to connect a care home MDS into such approaches—a top-down reorganisation to implement a dataset developed elsewhere, and in other contexts, is at odds with these approaches focussed around making the most of what is already collected, and empowerment and enfranchisement of localities and the people that live and work within them.

We faced issues with standardising approaches to data collection across two software providers and forty-five care homes. This variation in approach across different providers would be multiplied if the approaches described were rolled out to all 19 DCR providers accredited by NHSE. Plans underway by the Department of Health and Social Care to develop a Minimum Operating Data (MODS) standard [10] might facilitate some standardisation going forward. However, this MODS has been designed without the comprehensive evidence review and stakeholder consultation conducted for our pilot, and it contains a fraction of the variables included in our prototype MDS. It is likely to be, at best, an adjunct to a more comprehensive solution and will likely require iteration as implementation challenges of the sort described here unfold.

Our prototype MDS focussed on healthcare variables. This reflects, in part, the prominence given to these by all contributors, including care home staff and public representatives, during stakeholder work [16–18]. It also reflects the fact that routine healthcare data are often collected in a way that enables systematic collation and linkage. Healthcare data, by its nature, is aligned with standardised international approaches to coding care data, such as SNOMED and ICD-10. We found some data in DCRs stored as free text—an approach that provides nuanced and personalised records but hampers collation and analysis at meso- and macro-levels using standardised coding approaches. For now, there is a trade-off between data collatable in an MDS and data held in free-text. This may, though, be addressed by advances in machine-based analysis of free text in the future. Regardless, the integration of datasets across multiple sources represents an additional layer of challenge, as each source dataset may make its own changes over time. Keeping on top of these and the data manipulation and derivation required for a linked MDS has ongoing staffing resource requirements.

The incorporation of social care-related QoL and well-being, in the form of the ASCOT-Proxy-Resident and ICECAP-O measures, goes some way towards standardisation of data held in the social care record by providing person-centred data focussed around what matters to residents and relatives, collected in a standardised way. QoL data have been highlighted as essential for understanding quality in the sector [64].

We presented in an appendix how the MDS could facilitate understanding of care home residents’ use of ambulance services and hospital emergency departments. Our stakeholder work revealed other areas where an MDS could generate insights, including reasons for hospital admissions to inform local service provision or training needs and understanding pathways and access to services for residents with, for example, diabetes or mental health needs. Whilst this stakeholder wish list demonstrates the potential of an MDS to better understand resident needs, it also raises the challenge frequently reported in the care home literature of care home staff and providers feeling that they are at the mercy of external forces beyond their control [4, 12, 60, 65]. The evidence on what enables NHS services working with care homes to achieve improved outcomes consistently points to systems and practices that initiate and sustain quality working relationships between health and social care staff and their organisations [60, 66–70]. The powerful insights are deliverable through an MDS come with attendant responsibilities. Ensuring that data are used in a way that fosters trust between different stakeholder groups is an implementation imperative.

In conclusion, we have developed and demonstrated an MDS based on data linkage for English care homes. We have identified issues around data quality, information governance, plurality of data and the need for implementation approaches that facilitate data completion, which are essential to the implementation of any MDS in English care homes. We have also demonstrated the value of combining data sources to provide richer data and crucially reduce external requests for information from care homes. It is essential that this work moves forward to ensure that we can take data-informed approaches to care delivery, service design, commissioning and policy for the care home sector.

## Supplementary Material

aa-24-1229-File002_afaf001

## Data Availability

Anonymised data (digital care records and some associated variables) will be available on request from the corresponding author following a 24-month embargo from the date of publication.
