# Supplementary material for: Piloting a minimum data set for older people living in care homes in England: a developmental study
Source: Age Ageing. 2025 Jan 15;54(1):afaf001. doi: 10.1093/ageing/afaf001 (PMC11733825; doi:10.1093/ageing/afaf001)
Supplement: aa-24-1229-File002_afaf001 [file aa-24-1229-file002_afaf001.docx]

**APPENDIX 1 – Core Tenets of an MDS for long-term care homes, reproduced from Burton et al**[20]

| 1. The MDS must primarily focus on measuring what matters most to support those living in care homes through systematic data collection and sharing. 2. The MDS must be evidence-based in design and contents, requiring co-production with key stakeholders. 3. The MDS must reduce data burden and duplication of effort for the care home. This will be achieved through piloting, collaboration, and ongoing engagement with homes. 4. The MDS will be most effective when underpinned by digital care planning and care records systems, within the care home, serving the day-to-day needs of residents, staff, families, and friends. This requires digital infrastructure and investment to deliver at scale. 5. The MDS will include information on the care home service, individual-level data on residents, and information on the model of staffing that supports them, but will not include individual-level data identifying the workforce in each home. 6. The MDS should bring together data from within the care home, coupled with data held externally about residents and care services. 7. Data sharing with external users of the MDS must have an agreed purpose. Data sharing pathways must be defined and formalised in data sharing agreements, using secure environments for access where appropriate. Care home residents’ privacy rights must be protected. 8. Care homes should be supported to access and use the data they collect and share using electronic dashboards. 9. The MDS requires national infrastructure and integration with existing data systems. |
| --- |

**APPENDIX 2 – the Aspirational Minimum Data Set with Proposed Dataset as published in the initial study protocol** [11]

|  | Sections | Example variables | Digital care records | Health and social care datasets |
| --- | --- | --- | --- | --- |
| 1 | Demographics/characteristics | Date of birth; sex; NHS no; area-based deprivation | No | Personal demographics service |
|  |  | Religion, languages, marital or partnership status, deprivation of liberty | Yes | No |
|  |  | Ethnicity; weight; height | Yes | GP data; secondary user services data |
| 2 | Palliative care needs | End of life pathway register | No | GP data |
| 3 | Care home stay | Date of entry to care home; date of death | Yes | No |
| 4 | Resident needs | Skin condition | Yes | No |
|  |  | Cognitive impairment and impact on perception, understanding and need for support | Yes* | GP data; secondary user services data |
|  |  | Oral/nutritional status | No | Secondary user services data |
|  |  | Continence | No | Community datasets (where available) |
|  |  | Ability to perform activities of daily living; cognitive performance; delirium | Yes* | No |
| 5 | Quality of life | Outcomes; mood; dementia quality of life | Yes* | No |
| 6 | Complications/ adverse events | Infections | Yes | GP data; secondary user services data |
|  |  | Falls (leading to hospital admission or GP visit) | Yes | Secondary user services data; 999 data; ambulance data |
|  |  | Falls (only captured at care home level); early warning score; unintended weight loss | Yes | No |
| 7 | Diagnoses | Medical history | No | Secondary user services data; GP data |
|  |  | Frailty | No | GP data |
|  |  | Adverse reactions and allergies | No | GP data |
| 8 | Medication and vaccination | Prescribed medication and administered vaccines | No | GP data |
| 9 | Healthcare utilisation | Primary care use | No | GP data; NHS 111 data; 999 data |
|  |  | Community nursing; community allied health professionals | No | Community services data set |
|  |  | Out-of-hours contacts | No | GP out-of-hours data |
|  |  | Ambulance call-outs | No | Ambulance data |
|  |  | Accident and Emergency (A&E) attendance; emergency admissions; secondary care usage (outpatient appointments and elective admission) | No | Secondary user services data |
| 10 | Care home characteristics and workforce characteristics | Type of home; care home characteristics, specialities and client groups; location of care home; area-based deprivation; registered bed capacity; sector of provider; provider ownership type; CQC rating | No | CQC data |
|  |  | Staffing model; staffing ratios; numbers and types of staff; no of agency staff; no and type of vacancies | No† | Skills for care data |

***Added to the software for the purposes of the pilot study.**

**†As the Skills for Care workforce survey is voluntary, participating homes were asked to provide some information on workforce as part of a short online survey for the pilot.**

**CQC: Care Quality Commission; GP: general practitioner; MDS: minimum data set; NHS: National Health Service.**

**APPENDIX 3 - Data Flow Diagram**


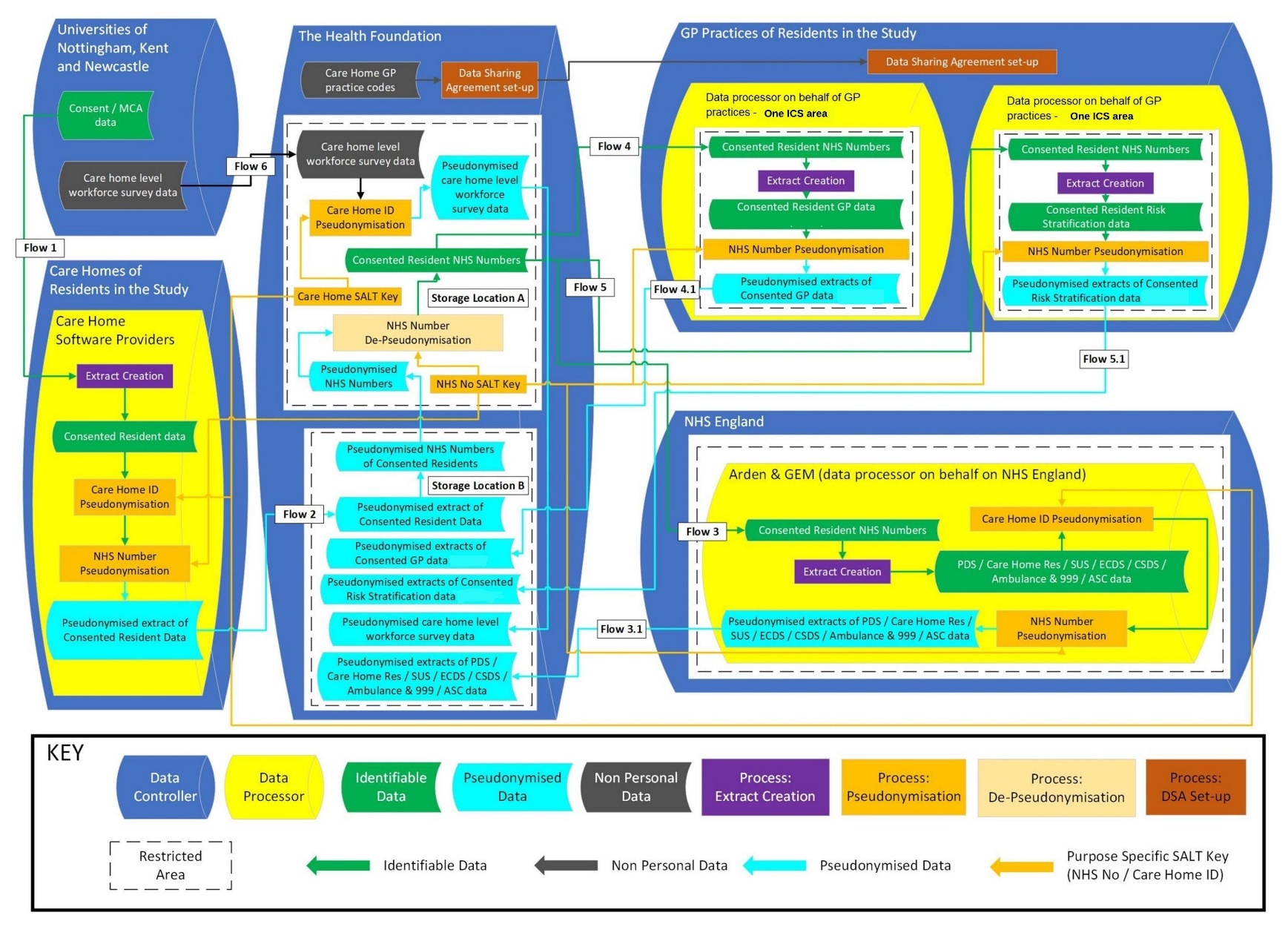


**APPENDIX 4 - Data Sharing Summary Diagram**


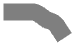

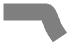


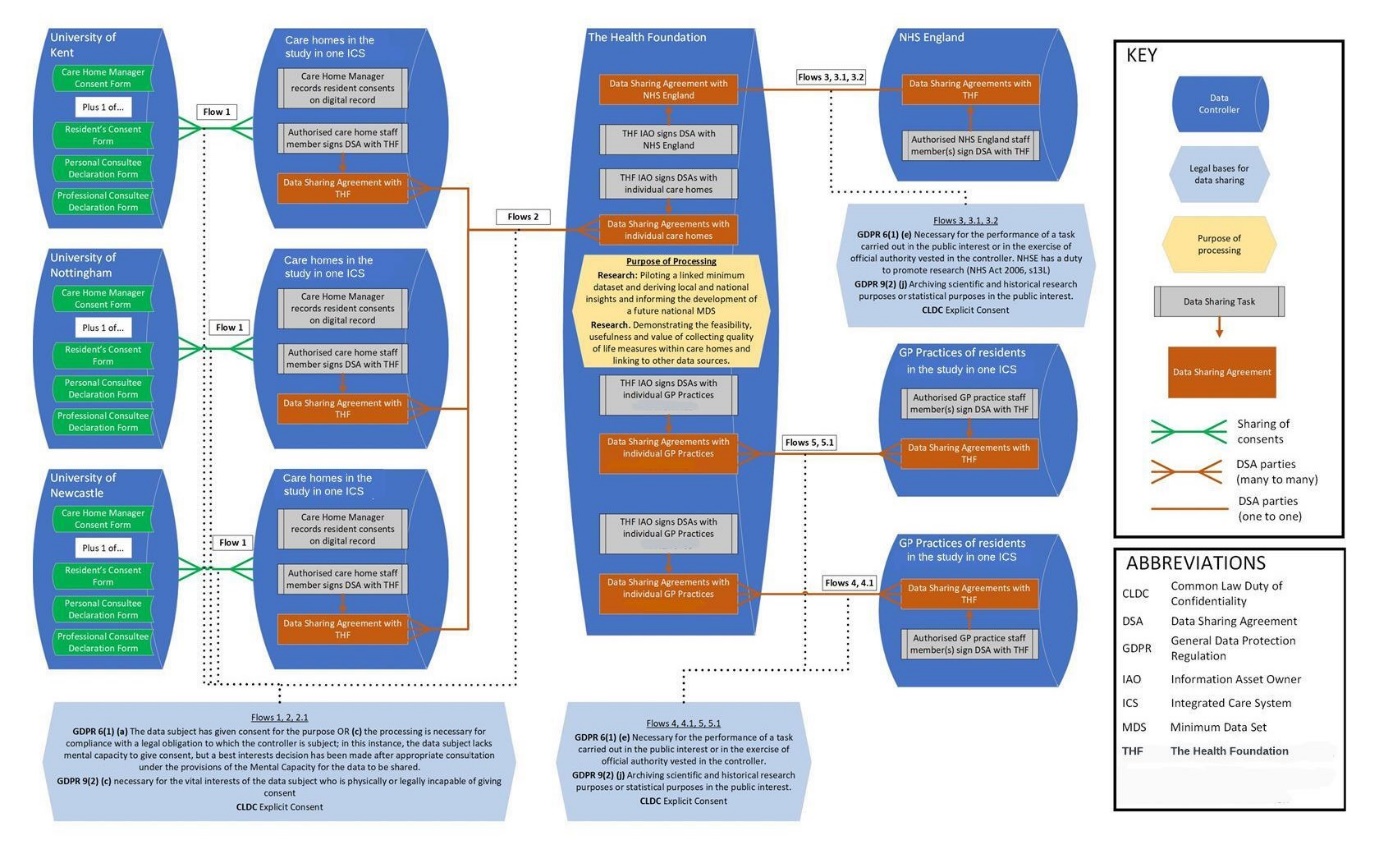


**APPENDIX 5 - Data specifications**

**Table a: Data specification for final prototype MDS**

| **Domain** | **Variable name** | **Description** | **Dataset^1^** | **Raw or derived** | **Derivation method** |
| --- | --- | --- | --- | --- | --- |
| Demographics/ characteristics | ethnicity_final | Ethnicity (final) | SUS, CSDS, DCR | Derived | From SUS APC, SUS OP and ECDS, mode was taken across all events for the patient (in patient episode, ED attendance or OP appointment).  Where there was no modus, counts were generated by patient and ethnicity category. The following code is applied if there is any record in a given category:  If there is any record as white and other records are not stated or missing, recorded as white.  If there is any record as black and other records are not stated or missing, recorded as black.  If there is any record as asian and other records are not stated or missing, recorded as asian.  If there is any record as mixed and other records are not stated or missing, recorded as mixed.  If there is any record as other and other records are not stated or missing, recorded as other.  If there is any record as not stated then recorded as not stated.  If none of the above, recorded as missing.  The ethnicity record was taken from DCR, then SUS if missing in DCR, then CSDS if missing in SUS. |
| Demographics/ characteristics | sex_final | Sex (final) | SUS, PDS, CSDS, DCR, | Derived | From SUS APC, SUS OP and ECDS, mode was taken across all events for the patient (in patient episode, ED attendance or OP appointment).  Sex was taken from PDS, then SUS if missing in PDS, then CSDS if missing in SUS, then DCR if missing in PDS. |
| Demographics/ characteristics | dob_final | Date of birth record | SUS, PDS | Derived | From SUS APC, SUS OP and SUS ECDS, mode was taken across all events for the patient (in patient episode, ED attendance or OP appointment). Date of birth was generated as the 1st of the month/year.  The date of birth was taken from PDS, then SUS if missing in PDS. |
| Demographics/ characteristics | dod_final | Date of death record | SUS, PDS, Care home residency | Derived | From hospital records, death date was taken from the latest of:  SUS APC (Episode End date where Discharge Method = 4) and ECDS (Departure Date where Discharge status = "75004002", or Treatment Date or Arrival Date is used if later)  Date of death was taken from PDS, then SUS if missing in PDS, then Care home residency if missing in SUS. |
| Demographics/ characteristics | religion_new | Religion | DCR | Derived | Derived variable. Coded differently by the two software providers. |
| Demographics/ characteristics | maritalstatus_new | Marital status | DCR | Raw | NA |
| Demographics/ characteristics | firstlanguage | First language spoken | DCR | Raw | NA |
| Demographics/ characteristics | powerattorney | Power of attorney | DCR | Raw | NA |
| Demographics/ characteristics | dols | Deprivation of Liberty status | DCR | Raw | NA |
| Demographics/ characteristics | dnacpr | DNACPR status | DCR | Raw | NA |
| Demographics/ characteristics | weight_band | Weight | DCR | Derived | Provider 1 numerical data converted to align with provider 2 categorical data |
| Demographics/ characteristics | height_band | Height | DCR | Derived | Provider 1 numerical data converted to align with provider 2 categorical data |
| Demographics/ characteristics | IMD_quintile | Indices of Deprivation 2019 quintile | PDS; ONS | Derived | LSOA sourced from PDS and combined with ONS Indices of Deprivation 2019 to identify deprivation quintile |
| Palliative care needs | DiscussedPreferredDeathLocation_Indicator | Discussed preferred death location indicator | CSDS | Raw | NA |
| Palliative care needs | DeathLocationPreferred_Type | Preferred death location | CSDS | Raw | NA |
| Care home stay | Client_Funding_Status_ASC | Client funding status | ASC | Raw | N/A |
| Care home stay | discharge_ch_12h | Discharge from an in-patient spell to a care home (1 year history) | SUS-APC | Derived | Discharge to care home was derived based on Discharge Destination (codes '54', '65') from continuous in-patient spells.^2^  Coded as 1 (yes) if there was any record of this in the year before the index date. |
| Care home stay | death_hosp_postindex | Death in hospital in the period between the index date and end of study. | SUS-APC | Derived | Death in hospital was derived based on Discharge Method (code '4') from continuous in-patient spells.^2^  Coded as 1 (yes) if there was any record of this between the index date and 31 October 2023. |
| Care home stay | los | Length of stay | DCR | Derived | Derived from date of entry to home. For Provider 2, only have month/year, so set to the 1st of the month. |
| Resident needs | mdscps | Cognitive impairment | DCR | Derived | The MDS CPS is calculated from five items: comatose, problem with short-term memory, cognitive skills for daily decision making, being understood by others, and eating ADL. Scored per Morris *et al*, 1994. |
| Resident needs | barthel_bowel | Bowel continence | DCR | Derived | Score from lowest (0) to highest (2) level of functional independence for each item. These are summed, x5, to create a score from lowest (0) to highest (100) independence. |
| Resident needs | barthel_bladder | Bladder continence | DCR | Derived | Score from lowest (0) to highest (2) level of functional independence for each item. These are summed, x5, to create a score from lowest (0) to highest (100) independence. |
| Resident needs | ascot_q11p | ASCOT: pain | DCR | Derived | Required some recoding to combine |
| Resident needs | ascot_mascore_p | ASCOT: anxiety and low mood | DCR | Derived | Required some recoding to combine |
| Resident needs | iddsi_food | Food texture requirements | DCR | Raw | NA |
| Resident needs | iddsi_drink | Drink thickness requirements | DCR | Raw | NA |
| Resident needs | allergy_food | Food allergy | DCR | Raw | NA |
| Resident needs | allergy_contact | Contact allergy | DCR | Raw | NA |
| Resident needs | allergy_med | Medication allergy | DCR | Raw | NA |
| Resident needs | allergy_penicillin | Penicillin allergy | DCR | Raw | NA |
| Resident needs | waterlow recent | Pressure ulcers (Waterlow score) | DCR | Raw | NA |
| Resident needs | braden | Pressure ulcers (Braden score) | DCR | Raw | NA |
| Resident needs | iaged score | Delirium (I-AGED score) | DCR | Derived | Each of the ten items is scored no (0) or yes (1) and summed to create a scale from 0 to 10. |
| Resident needs | barthel | Functional independence (Barthel score) | DCR | Derived | Score from lowest (0) to highest (2) level of functional independence for each item. These are summed, x5, to create a score from lowest (0) to highest (100) independence. |
| Resident needs | n_ED_attendances_ngproc | Emergency department attendances with nasogastric procedure (1 year history) | SUS-ECDS | Derived | Coded as 1 (yes) if there was a SNOMED code for nasogastric procedure from Der_EC_Treatment_All ("87750000", "6125005", "112861000") in the year before index date, otherwise coded as 0 (no) |
| Resident needs | n_ngproc_op | Outpatient appointments with nasogastric procedure (1 year history) | SUS-OP | Derived | Coded as 1 (yes) if there was a procedure code for nasogastric procedure from Der_Procedure_All (OPCS codes) recorded for an outpatient appointment in the year before index date, otherwise coded as 0 (no) |
| Resident needs | mdscps | Cognitive impairment | DCR | Derived | MDS CPS is calculated from five items: comatose, problem with short-term memory, cognitive skills for daily decision making, being understood by others, and eating ADL. Scored per Morris *et al*, 1994. |
| Quality of life | ascs_qol_score | Quality of life overall | DCR | Derived | Required some recoding to combine |
| Quality of life | ascot_scrqol | Ascot Proxy-Resident | DCR | Derived | Required some recoding to combine. Applied preference weights for ASCOT SCT4 to generate index score from -.17 to 1.0. |
| Quality of life | icecap_qol | ICECAP-O | DCR | Derived | Required some recoding to combine. Score (0 to 1) calculated using UK index values [. |
| Quality of life | UK_crosswalk | EQ-5D-5L Proxy | DCR | Derived | Required some recoding to combine.  Score calculated using the mapping function to convert to EQ-5D-3L and applied UK index values. The UK value set for the EQ-5D-5L is still being developed [45,46] |
| Complications/ adverse events | must_score_recent | MUST (malnutrition universal scoring tool) score | DCR | Raw | NA |
| Complications/ adverse events | adm_lrti_12h | Frequency of in-patient admissions with lower respiratory tract infection recorded (1 year history) | SUS-APC | Derived | Admissions with lrti recorded were derived based on ICD-10 codes in the primary diagnosis field .  Count of admissions with lrti recorded from continuous in-patient spells in the year before the index date.^2^ |
| Complications/ adverse events | adm_urti_12h | Frequency of in-patient admissions with upper respiratory tract infection recorded (1 year history) | SUS-APC | Derived | Admissions with urti recorded were derived based on ICD-10 codes in the primary diagnosis field.  Count of admissions with urti recorded from continuous in-patient spells in the year before the index date.^2^ |
| Diagnoses | cogimp_sus | Cognitive impairment (delirium, dementia, senility) | SUS-APC | Derived | Diagnosis flags were derived based on ICD-10 codes in the diagnosis fields for each episode.  Coded as 1 (yes) if there was a record of any of delirium, dementia or senility (using Soong et al 2015 code list) in the patient's hospital admissions in 3 years before the index date, otherwise coded as 0 (no) |
| Diagnoses | dementia_final | Dementia (final) | SUS-APC and DCR | Derived | Diagnosis flags were derived from SUS-APC based on ICD-10 codes in the diagnosis fields for each episode.  Dementia status is selected first from SUS (a resident has dementia if identified through either the code list used for Charlson index or Soong et al Frailty), coded as 1 if there was any record of the condition in the patient's hospital admissions in the previous 3 years, and if missing, from DCR. Note if a person does not have dementia recorded in SUS but does in care home record then they will be recorded as having dementia in dementia_final. |
| Diagnoses | nr_elix_h36 | Elixhauser no conditions based on 3 years history | SUS-APC | Derived | Diagnosis flags were derived based on ICD-10 codes in the diagnosis fields for each episode.  Count of conditions in the Elixhauser list of comorbidities^3^ in the patient's hospital admissions in 3 years before the index date. |
| Diagnoses | nr_elix_2_h36 | Elixhauser conditions >=2 based on 3 years history | SUS-APC | Derived | Diagnosis flags were derived based on ICD-10 codes in the diagnosis fields for each episode.  Coded as 1 (yes) if there were >= conditions in the Elixhauser list of comorbidities^3^ in the patient's hospital admissions in 3 years before the index date. |
| Diagnoses | e_alco_abuse_h36 | Alcohol abuse^2^ | SUS-APC | Derived | Diagnosis flags were derived based on ICD-10 codes in the diagnosis fields for each episode.  Coded as 1 (yes) if there was any record of the condition in the patient's hospital admissions in 3 years before the index date. |
| Diagnoses | e_anaemia_bloodloss_h36 | Blood loss anaemia^2^ | SUS-APC | Derived | Diagnosis flags were derived based on ICD-10 codes in the diagnosis fields for each episode.  Coded as 1 (yes) if there was any record of the condition in the patient's hospital admissions in 3 years before the index date. |
| Diagnoses | e_anaemia_deficiency_h36 | Deficiency anaemia^2^ | SUS-APC | Derived | Diagnosis flags were derived based on ICD-10 codes in the diagnosis fields for each episode.  Coded as 1 (yes) if there was any record of the condition in the patient's hospital admissions in 3 years before the index date. |
| Diagnoses | e_arrhythmias_h36 | Cardiac arrhythmias^2^ | SUS-APC | Derived | Diagnosis flags were derived based on ICD-10 codes in the diagnosis fields for each episode.  Coded as 1 (yes) if there was any record of the condition in the patient's hospital admissions in 3 years before the index date. |
| Diagnoses | e_coagulopathy_h36 | Coagulopathy^2^ | SUS-APC | Derived | Diagnosis flags were derived based on ICD-10 codes in the diagnosis fields for each episode.  Coded as 1 (yes) if there was any record of the condition in the patient's hospital admissions in 3 years before the index date. |
| Diagnoses | e_depression_h36 | Depression^2^ | SUS-APC | Derived | Diagnosis flags were derived based on ICD-10 codes in the diagnosis fields for each episode.  Coded as 1 (yes) if there was any record of the condition in the patient's hospital admissions in 3 years before the index date. |
| Diagnoses | e_diab_comp_h36 | Diabetes, complicated^2^ | SUS-APC | Derived | Diagnosis flags were derived based on ICD-10 codes in the diagnosis fields for each episode.  Coded as 1 (yes) if there was any record of the condition in the patient's hospital admissions in 3 years before the index date. |
| Diagnoses | e_diab_uncomp_h36 | Diabetes, uncomplicated^2^ | SUS-APC | Derived | Diagnosis flags were derived based on ICD-10 codes in the diagnosis fields for each episode.  Coded as 1 (yes) if there was any record of the condition in the patient's hospital admissions in 3 years before the index date. |
| Diagnoses | e_drug_abuse_h36 | Drug abuse^2^ | SUS-APC | Derived | Diagnosis flags were derived based on ICD-10 codes in the diagnosis fields for each episode.  Coded as 1 (yes) if there was any record of the condition in the patient's hospital admissions in 3 years before the index date. |
| Diagnoses | e_fluid_h36 | Fluid and electrolyte disorders^2^ | SUS-APC | Derived | Diagnosis flags were derived based on ICD-10 codes in the diagnosis fields for each episode.  Coded as 1 (yes) if there was any record of the condition in the patient's hospital admissions in 3 years before the index date. |
| Diagnoses | e_ht_comp_h36 | Hypertension, complicated^2^ | SUS-APC | Derived | Diagnosis flags were derived based on ICD-10 codes in the diagnosis fields for each episode.  Coded as 1 (yes) if there was any record of the condition in the patient's hospital admissions in 3 years before the index date. |
| Diagnoses | e_ht_uncomp_h36 | Hypertension, uncomplicated^2^ | SUS-APC | Derived | Diagnosis flags were derived based on ICD-10 codes in the diagnosis fields for each episode.  Coded as 1 (yes) if there was any record of the condition in the patient's hospital admissions in 3 years before the index date. |
| Diagnoses | e_hypothyroid_h36 | Hypothyroidism^2^ | SUS-APC | Derived | Diagnosis flags were derived based on ICD-10 codes in the diagnosis fields for each episode.  Coded as 1 (yes) if there was any record of the condition in the patient's hospital admissions in 3 years before the index date. |
| Diagnoses | e_liver_h36 | Liver disease^2^ | SUS-APC | Derived | Diagnosis flags were derived based on ICD-10 codes in the diagnosis fields for each episode.  Coded as 1 (yes) if there was any record of the condition in the patient's hospital admissions in 3 years before the index date. |
| Diagnoses | e_lymphoma_h36 | Lymphoma^2^ | SUS-APC | Derived | Diagnosis flags were derived based on ICD-10 codes in the diagnosis fields for each episode.  Coded as 1 (yes) if there was any record of the condition in the patient's hospital admissions in 3 years before the index date. |
| Diagnoses | e_obesity_h36 | Obesity^2^ | SUS-APC | Derived | Diagnosis flags were derived based on ICD-10 codes in the diagnosis fields for each episode.  Coded as 1 (yes) if there was any record of the condition in the patient's hospital admissions in 3 years before the index date. |
| Diagnoses | e_other_neuro_h36 | Other neurological disorders^2^ | SUS-APC | Derived | Diagnosis flags were derived based on ICD-10 codes in the diagnosis fields for each episode.  Coded as 1 (yes) if there was any record of the condition in the patient's hospital admissions in 3 years before the index date. |
| Diagnoses | e_peptic_nobld_h36 | Peptic ulcer disease excl bleeding^2^ | SUS-APC | Derived | Diagnosis flags were derived based on ICD-10 codes in the diagnosis fields for each episode.  Coded as 1 (yes) if there was any record of the condition in the patient's hospital admissions in 3 years before the index date. |
| Diagnoses | e_psychoses_h36 | Psychoses^2^ | SUS-APC | Derived | Diagnosis flags were derived based on ICD-10 codes in the diagnosis fields for each episode.  Coded as 1 (yes) if there was any record of the condition in the patient's hospital admissions in 3 years before the index date. |
| Diagnoses | e_pulmcirc_h36 | Pulmonary circulation disorders^2^ | SUS-APC | Derived | Diagnosis flags were derived based on ICD-10 codes in the diagnosis fields for each episode.  Coded as 1 (yes) if there was any record of the condition in the patient's hospital admissions in 3 years before the index date. |
| Diagnoses | e_pvd_h36 | Peripheral vascular disease^2^ | SUS-APC | Derived | Diagnosis flags were derived based on ICD-10 codes in the diagnosis fields for each episode.  Coded as 1 (yes) if there was any record of the condition in the patient's hospital admissions in 3 years before the index date. |
| Diagnoses | e_renalfail_h36 | Rheumatoid arthritis / collagen vascular diseases^2^ | SUS-APC | Derived | Diagnosis flags were derived based on ICD-10 codes in the diagnosis fields for each episode.  Coded as 1 (yes) if there was any record of the condition in the patient's hospital admissions in 3 years before the index date. |
| Diagnoses | e_rheum_arth_h36 | Renal failure^2^ | SUS-APC | Derived | Diagnosis flags were derived based on ICD-10 codes in the diagnosis fields for each episode.  Coded as 1 (yes) if there was any record of the condition in the patient's hospital admissions in 3 years before the index date. |
| Diagnoses | e_stumour_nomets_h36 | Solid tumour without metastasis^2^ | SUS-APC | Derived | Diagnosis flags were derived based on ICD-10 codes in the diagnosis fields for each episode.  Coded as 1 (yes) if there was any record of the condition in the patient's hospital admissions in 3 years before the index date. |
| Diagnoses | e_valvular_h36 | Valvular disease^2^ | SUS-APC | Derived | Diagnosis flags were derived based on ICD-10 codes in the diagnosis fields for each episode.  Coded as 1 (yes) if there was any record of the condition in the patient's hospital admissions in 3 years before the index date. |
| Diagnoses | e_weight_loss_h36 | Weight loss^2^ | SUS-APC | Derived | Diagnosis flags were derived based on ICD-10 codes in the diagnosis fields for each episode.  Coded as 1 (yes) if there was any record of the condition in the patient's hospital admissions in 3 years before the index date. |
| Diagnoses | ec_chf_h36 | Congestive heart failure^2^ | SUS-APC | Derived | Diagnosis flags were derived based on ICD-10 codes in the diagnosis fields for each episode.  Coded as 1 (yes) if there was any record of the condition in the patient's hospital admissions in 3 years before the index date. |
| Diagnoses | ec_cpd_h36 | Chronic pulmonary disease^2^ | SUS-APC | Derived | Diagnosis flags were derived based on ICD-10 codes in the diagnosis fields for each episode.  Coded as 1 (yes) if there was any record of the condition in the patient's hospital admissions in 3 years before the index date. |
| Diagnoses | ec_plegia_h36 | Hemiplegia / paraplegia^2^ | SUS-APC | Derived | Diagnosis flags were derived based on ICD-10 codes in the diagnosis fields for each episode.  Coded as 1 (yes) if there was any record of the condition in the patient's hospital admissions in 3 years before the index date. |
| Diagnoses | ec_stumour_mets_h36 | Metastatic solid tumour / metastatic cancer^2^ | SUS-APC | Derived | Diagnosis flags were derived based on ICD-10 codes in the diagnosis fields for each episode.  Coded as 1 (yes) if there was any record of the condition in the patient's hospital admissions in 3 years before the index date. |
| Diagnoses | N frailty conditions | Number of frailty conditions | SUS-APC | Derived | Diagnosis flags were derived based on ICD-10 codes in the diagnosis fields for each episode.  Frailty syndromes were cognitive impairment, anxiety or depression, functional dependence, falls or fractures, incontinence, mobility problems and pressure ulcers (Soong et al 2015).  Count of frailty syndromes^4^ recorded in the patient's hospital admission in 3 years before the index date. |
| Diagnoses | f_delirium_h36 | Delirium^3^ | SUS-APC | Derived | Diagnosis flags were derived based on ICD-10 codes in the diagnosis fields for each episode.  Coded as 1 (yes) if there was any record of the condition in the patient's hospital admissions in 3 years before the index date. |
| Diagnoses | f_dementia_h36 | Dementia^3^ | SUS-APC | Derived | Diagnosis flags were derived based on ICD-10 codes in the diagnosis fields for each episode.  Coded as 1 (yes) if there was any record of the condition in the patient's hospital admissions in 3 years before the index date. |
| Diagnoses | f_senility_h36 | Senility^3^ | SUS-APC | Derived | Diagnosis flags were derived based on ICD-10 codes in the diagnosis fields for each episode.  Coded as 1 (yes) if there was any record of the condition in the patient's hospital admissions in 3 years before the index date. |
| Diagnoses | f_anxdep_h36 | Anxiety/Depression^3^ | SUS-APC | Derived | Diagnosis flags were derived based on ICD-10 codes in the diagnosis fields for each episode.  Coded as 1 (yes) if there was any record of the condition in the patient's hospital admissions in 3 years before the index date. |
| Diagnoses | f_dependence_h36 | Functional dependence^3^ | SUS-APC | Derived | Diagnosis flags were derived based on ICD-10 codes in the diagnosis fields for each episode.  Coded as 1 (yes) if there was any record of the condition in the patient's hospital admissions in 3 years before the index date. |
| Diagnoses | f_fallsfract_h36 | Falls/Fractures^3^ | SUS-APC | Derived | Diagnosis flags were derived based on ICD-10 codes in the diagnosis fields for each episode.  Coded as 1 (yes) if there was any record of the condition in the patient's hospital admissions in 3 years before the index date. |
| Diagnoses | f_incont_h36 | Incontinence^3^ | SUS-APC | Derived | Diagnosis flags were derived based on ICD-10 codes in the diagnosis fields for each episode.  Coded as 1 (yes) if there was any record of the condition in the patient's hospital admissions in 3 years before the index date. |
| Diagnoses | f_mobprob_h36 | Mobility problems^3^ | SUS-APC | Derived | Diagnosis flags were derived based on ICD-10 codes in the diagnosis fields for each episode.  Coded as 1 (yes) if there was any record of the condition in the patient's hospital admissions in 3 years before the index date. |
| Diagnoses | f_pulcers_h36 | Pressure ulcers^3^ | SUS-APC | Derived | Diagnosis flags were derived based on ICD-10 codes in the diagnosis fields for each episode.  Coded as 1 (yes) if there was any record of the condition in the patient's hospital admissions in 3 years before the index date. |
| Healthcare utilisation | adm_el_12h | Frequency of elective admissions (1 year history) | SUS-APC | Derived | Elective admissions were derived based on Admission Method indicating elective admission (codes '11','12','13').  Count of elective admissions from continuous in-patient spells in the year before the index date.^2^ |
| Healthcare utilisation | adm_em_12h | Frequency of emergency admissions (1 year history) | SUS-APC | Derived | Emergency admissions were derived based on Admission Method indicating Emergency Admission (codes '21','22','23','24','25','28','2A','2B','2C','2D').  Count of emergency admissions from continuous in-patient spells in the year before the index date.^2^ |
| Healthcare utilisation | adm_avoid_12h | Frequency of potentially avoidable emergency admissions (1 year history)^5^ | SUS-APC | Derived | Potentially avoidable emergency admissions were defined as emergency admissions (Admission Method codes '21','22','23','24','25','28','2A','2B','2C','2D') with one of a list of conditions as the primary diagnoses for the first episode of the hospital spell.  Count of potentially avoidable emergency admissions from continuous in-patient spells in the year before the index date.^2,5^ |
| Healthcare utilisation | n_ED_attendances | Frequency of emergency department attendances (1 year history) | SUS-ECDS | Derived | Count of non-duplicate ED attendances in 1 year before the index date |
| Healthcare utilisation | n_ED_attendances_via_ambulance | Frequency of emergency department attendances via ambulance (1 year history) | SUS-ECDS | Derived | Count of non-duplicate ED attendances that were via ambulance (as indicated by presence of codes 104803100000010, 1048021000000102, 1048041000000109, 1048051000000107 in field EC_Arrival_Mode_SNOMED_CT) in 1 year before the index date |
| Healthcare utilisation | n_out_of_hours_ED_attendances | Frequency of emergency department attendances after 6pm/ before 8am (1 year history) | SUS-ECDS | Derived | Count of non-duplicate ED attendances that occurred after 1800 or before 0800 hours in 1 year before the index date |
| Healthcare utilisation | n_long_ED_attendances | Frequency of emergency department attendances lasting more than 12 hours (1 year history) | SUS-ECDS | Derived | Count of non-duplicate ED attendances that lasted more than 12 hours in duration in 1 year before the index date |
| Healthcare utilisation | n_ED_attendances_for_falls | Frequency of emergency department attendances due to a fall (1 year history) | SUS-ECDS | Derived | Count of non-duplicate ED attendances that were for falls (as indicated by presence of codes 161898004, 430576002, 54670004, 75941004, 240871000000104, 429482004 in field EC_Chief_Complaint_SNOMED_CT) in 1 year before the index date |
| Healthcare utilisation | n_OP_appts | Frequency of outpatient appointments (1 year history) | SUS-OP | Derived | Count of non-duplicate outpatient appointments in 1 year before index date |
| Healthcare utilisation | n_cs_appts | Frequency of community services appointments (1 year history) | CSDS | Derived | Count of community service appointments in 1 year before the index date |
| Healthcare utilisation | n_missed_OP_appts | Frequency of missed outpatient appointments (1 year history) | SUS-OP | Derived | Count of non-duplicate appointments that were missed (as indicated by presence of codes 0, 1, 3 in field Attendance_Status) in 1 year before the index date |
| Healthcare utilisation | n_cs_appts_continence | Frequency of continence appointments (1 year history) | CSDS | Derived | Count of continence community service appointments (as indicated by Referral_TeamType =07) in 1 year before the index date |
| Healthcare utilisation | n_cs_appts_dn | Frequency of district nursing appointments (1 year history) | CSDS | Derived | Count of district nursing community service appointments (as indicated by Referral_TeamType =12) in 1 year before the index date |
| Healthcare utilisation | n_cs_appts_podiatry | Frequency of podiatry appointments (1 year history) | CSDS | Derived | Count of podiatry community service appointments (as indicated by Referral_TeamType =27) in 1 year before the index date |
| Healthcare utilisation | n_cs_appts_rehab | Frequency of community rehabilitation appointments (1 year history) | CSDS | Derived | Count of community rehabilitation community service appointments (as indicated by Referral_TeamType =29) in 1 year before the index date |
| Healthcare utilisation | n_cs_appts_SALT | Frequency of speech and language therapy appointments (1 year history) | CSDS | Derived | Count of SALT community service appointments (as indicated by Referral_TeamType =33) in 1 year before the index date |
| Healthcare utilisation | n_cs_f2f_appts | Frequency of face to face community services appointments (1 year history) | CSDS | Derived | Count of face to face community service appointments (as indicated by presence of code 1 in field Consultation_MethodUsed) in 1 year before the index date |
| Healthcare utilisation | n_cs_missed_appts | Frequency of missed community services appointments (1 year history) | CSDS | Derived | Count of missed community service appointments (as indicated by presence of codes 2, 3, 7 in field AttendanceStatus) in 1 year before the index date |
| Healthcare utilisation | n_cs_services | Number of community services seen (1 year history) | CSDS | Derived | Count of community service teams seen in 1 year before the index date |
| Healthcare utilisation | n_ambulance_call_outs_5m | Frequency of ambulance call outs (1 June-31 October 2023) | Ambulance | Derived | Count of ambulance call outs between 1 June 2023 and 31 October 2023 |
| Healthcare utilisation | n_ambulance_attendances_5m | Frequency of ambulance attendances (1 June-31 October 2023) | Ambulance | Derived | Count of ambulance attendances between 1 June 2023 and 31 October 2023 |
| Healthcare utilisation | n_ambulance_attendances_OOH_5m | Frequency of ambulance attendances after 6pm/ before 8am (1 June-31 October 2023) | Ambulance | Derived | Count of ambulance attendances that occurred after 1800 or before 0800 hours between 1 June 2023 and 31 October 2023 |
| Healthcare utilisation | n_ambulance_conveyances_5m | Frequency of ambulance conveyances (1 June-31 October 2023) | Ambulance | Derived | Count of ambulance conveyances between 1 June 2023 and 31 October 2023 |
| Care home characteristics and workforce characteristics | Der_CQC_Service_Type_CHR | Service type | Care home residency | Raw | NA |
| Care home characteristics and workforce characteristics | registered_bed_capacity | Registered bed capacity | CQC | Derived | Numeric converted into categorical variable |
| Care home characteristics and workforce characteristics | Rating_overall | CQC rating (overall) | CQC | Raw | NA |
| Care home characteristics and workforce characteristics | service_reg_yrs | Years since registration with CQC | CQC | Derived | Numeric converted into categorical variable |
| Care home characteristics and workforce characteristics | Q3_1_4 | Number of beds | Care home survey | Raw | NA |
| Care home characteristics and workforce characteristics | Q3_2_4 | Number of beds currently occupied | Care home survey | Raw | NA |
| Care home characteristics and workforce characteristics | Q3_3_4 | Number of resident fully self-funding | Care home survey | Raw | NA |
| Care home characteristics and workforce characteristics | Q3_4_4 | Number of staff | Care home survey | Raw | NA |
| Care home characteristics and workforce characteristics | Q3_5_4 | Number of full-time staff | Care home survey | Raw | NA |
| Care home characteristics and workforce characteristics | Q3_6_4 | Number of part-time staff | Care home survey | Raw | NA |
| Care home characteristics and workforce characteristics | Q3_7_4 | Number of staff on permanent contracts | Care home survey | Raw | NA |
| Care home characteristics and workforce characteristics | Q3_8_4 | Number of staff vacancies | Care home survey | Raw | NA |
| Care home characteristics and workforce characteristics | Q3_9_4 | Number of agency staff | Care home survey | Raw | NA |
| Care home characteristics and workforce characteristics | Q3_10_4 | Number of care workers | Care home survey | Raw | NA |
| Care home characteristics and workforce characteristics | Q3_11_4 | Number of senior care workers | Care home survey | Raw | NA |
| Care home characteristics and workforce characteristics | Q3_13_4 | Number of registered nurses | Care home survey | Raw | NA |
| Care home characteristics and workforce characteristics | Q3_12_4 | Number of nursing associates | Care home survey | Raw | NA |
| Care home characteristics and workforce characteristics | Q3_14_4 | Number of nursing assistants | Care home survey | Raw | NA |
| Care home characteristics and workforce characteristics | Q3_15_4 | Number of allied health professionals | Care home survey | Raw | NA |
| Care home characteristics and workforce characteristics | Q3_16_4 | Number of activities coordinators | Care home survey | Raw | NA |
| Care home characteristics and workforce characteristics | Q3_17_4 | Number of staff in roles above on permanent contracts | Care home survey | Raw | NA |
| Care home characteristics and workforce characteristics | Q3_18_4 | Number of staff vacancies in roles above | Care home survey | Raw | NA |
| Care home characteristics and workforce characteristics | Q3_19_4 | Number of agency staff in roles above | Care home survey | Raw | NA |

^1^ Dataset abbreviations: ASC-CLD = Adult Social Care Client Level Dataset; CQC = Care Quality Commision; CSDS = Community Services Data Set; DCR = Digital Care Record; ONS = Office for National Statistics; PDS = Personal Demographics Service; SUS = Secondary Uses Service; SUS-APC = SUS Admitted Patient Care; SUS-ECDS = SUS Emergency Care Dataset; SUS-OP = SUS Outpatient.

^2^ Continuous in-patient spells (CIPS) (sequence of spells from patient's first admission to hospital to patient final discharge home, including transfers to other hospitals as part of patient's care) were derived by grouping episodes with the same patient, provider and admission date (or previous episode end date the same as the following episode start date).

^3^ From the list of Elixhauser conditions (M Elixhauser A, Steiner C, Harris DR, et al. Comorbidity measures for use with administrative data. Med Care. 1998;36:8–27. doi: 10.1097/00005650-199801000-00004; Quan H, Sundararajan V, Halfon P, et al. Coding algorithms for defining comorbidities in ICD-9-CM and ICD-10 administrative data. Med Care. 2005;43:1130–9. doi: 10.1097/01.MLR.0000182534.19832.83.)

^4^ From a validated list of frailty syndromes (Soong J, Poots AJ, Scott S, et al. Developing and validating a risk prediction model for acute care based on frailty syndromes. BMJ Open. 2015;5. doi: 10.1136/BMJOPEN-2015-008457)

^5^ Potentially avoidable emergency admissions (Care Quality Commission, Great Britain. Parliament. House of Commons. The state of health care and adult social care in England in 2012/13. 2013;86.).

**Table b: data dictionary for linked routinely collected source datasets**

| **Dataset^1^** | **Domain** | **Variable name** | **Description** |
| --- | --- | --- | --- |
| ALL | Linkage | pseudonhsno | Pseudonymised NHS number |
| SUS-APCE | Healthcare utilization | Generated_Record_ID | Generated Record ID |
| SUS-APCE | Healthcare utilization | Der_Postcode_LSOA_Code | LSOA code |
| SUS-APCE | Healthcare utilization | GP_Practice_Code | GP practice code |
| SUS-APCE | Healthcare utilization | Der_Postcode_CCG_Code | CCG code |
| SUS-APCE | Healthcare utilization | Der_Financial_Year | Financial year |
| SUS-APCE | Healthcare utilization | Der_Activity_Month | Activity month |
| SUS-APCE | Healthcare utilization | Admission_Date | Admission date |
| SUS-APCE | Healthcare utilization | Admission_Time | Admission time |
| SUS-APCE | Healthcare utilization | Admission_Method | Admission method |
| SUS-APCE | Healthcare utilization | Source_of_Admission | Source of admission |
| SUS-APCE | Healthcare utilization | Administrative_Category | Administrative category |
| SUS-APCE | Healthcare utilization | Discharge_Date | Discharge date |
| SUS-APCE | Healthcare utilization | Discharge_Time | Discharge time |
| SUS-APCE | Healthcare utilization | Discharge_Method | Discharge method |
| SUS-APCE | Healthcare utilization | Discharge_Destination | Discharge destination |
| SUS-APCE | Healthcare utilization | Der_Provider_Code | Provider code |
| SUS-APCE | Diagnoses | Der_Diagnosis_Count | Count of diagnosis codes |
| SUS-APCE | Diagnoses | Der_Primary_Diagnosis_Code | Primary diagnosis code |
| SUS-APCE | Diagnoses | Der_Secondary_Diagnosis_Code_1 | Secondary diagnosis code 1 |
| SUS-APCE | Diagnoses | Der_Secondary_Diagnosis_Code_2 | Secondary Diagnosis Code 2 |
| SUS-APCE | Diagnoses | Der_Secondary_Diagnosis_Code_3 | Secondary Diagnosis Code 3 |
| SUS-APCE | Diagnoses | Der_Secondary_Diagnosis_Code_4 | Secondary Diagnosis Code 4 |
| SUS-APCE | Diagnoses | Der_Secondary_Diagnosis_Code_5 | Secondary Diagnosis Code 5 |
| SUS-APCE | Diagnoses | Der_Secondary_Diagnosis_Code_6 | Secondary Diagnosis Code 6 |
| SUS-APCE | Diagnoses | Der_Secondary_Diagnosis_Code_7 | Secondary Diagnosis Code 7 |
| SUS-APCE | Diagnoses | Der_Secondary_Diagnosis_Code_8 | Secondary Diagnosis Code 8 |
| SUS-APCE | Diagnoses | Der_Secondary_Diagnosis_Code_9 | Secondary Diagnosis Code 9 |
| SUS-APCE | Diagnoses | Der_Secondary_Diagnosis_Code_10 | Secondary Diagnosis Code 10 |
| SUS-APCE | Diagnoses | Der_Secondary_Diagnosis_Code_11 | Secondary Diagnosis Code 11 |
| SUS-APCE | Diagnoses | Der_Secondary_Diagnosis_Code_12 | Secondary Diagnosis Code 12 |
| SUS-APCE | Diagnoses | Der_Secondary_Diagnosis_Code_13 | Secondary Diagnosis Code 13 |
| SUS-APCE | Diagnoses | Der_Secondary_Diagnosis_Code_14 | Secondary Diagnosis Code 14 |
| SUS-APCE | Diagnoses | Der_Secondary_Diagnosis_Code_15 | Secondary Diagnosis Code 15 |
| SUS-APCE | Diagnoses | Der_Secondary_Diagnosis_Code_16 | Secondary Diagnosis Code 16 |
| SUS-APCE | Diagnoses | Der_Secondary_Diagnosis_Code_17 | Secondary Diagnosis Code 17 |
| SUS-APCE | Diagnoses | Der_Secondary_Diagnosis_Code_18 | Secondary Diagnosis Code 18 |
| SUS-APCE | Diagnoses | Der_Secondary_Diagnosis_Code_19 | Secondary Diagnosis Code 19 |
| SUS-APCE | Diagnoses | Der_Secondary_Diagnosis_Code_20 | Secondary Diagnosis Code 20 |
| SUS-APCE | Diagnoses | Der_Secondary_Diagnosis_Code_21 | Secondary Diagnosis Code 21 |
| SUS-APCE | Diagnoses | Der_Secondary_Diagnosis_Code_22 | Secondary Diagnosis Code 22 |
| SUS-APCE | Diagnoses | Der_Secondary_Diagnosis_Code_23 | Secondary Diagnosis Code 23 |
| SUS-APCE | Diagnoses | Der_Diagnosis_All | All diagnosis codes |
| SUS-APCE | Healthcare utilization | Treatment_Function_Code | Treatment function code |
| SUS-APCE | Healthcare utilization | Main_Speciality_Code | Main speciality code |
| SUS-APCE | Healthcare utilization | Patient_Classification | Patient classification |
| SUS-APCE | Healthcare utilization | Episode_Start_Date | Episode Start Date |
| SUS-APCE | Healthcare utilization | Episode_End_Date | Episode End Date |
| SUS-APCE | Healthcare utilization | Der_Episode_Number | Episode Number |
| SUS-APCE | Healthcare utilization | Der_Spell_ID | Spell ID |
| SUS-APCE | Demographics/characteristics | Month_of_Birth_SUS | Month of birth |
| SUS-APCE | Demographics/characteristics | Year_of_Birth_SUS | Year of birth |
| SUS-APCE | Demographics/characteristics | Sex | Sex |
| SUS-APCE | Demographics/characteristics | Ethnic_Group | Ethnicity |
| SUS-ECDS | Healthcare utilization | Arrival_Date | Date of arrival |
| SUS-ECDS | Healthcare utilization | Arrival_Time | Time of arrival |
| SUS-ECDS | Healthcare utilization | EC_Treatment_Date_01 | Date of treatment |
| SUS-ECDS | Healthcare utilization | EC_Seen_For_Treatment_Time_Since_Arrival | Waiting time from arrival to being seen for treatment |
| SUS-ECDS | Healthcare utilization | EC_Departure_Date | Date of departure |
| SUS-ECDS | Healthcare utilization | EC_Departure_Time | Time of departure |
| SUS-ECDS | Healthcare utilization | Der_EC_Duration | Duration of the A&E attendance |
| SUS-ECDS | Healthcare utilization | Der_Activity_Month | Activity month |
| SUS-ECDS | Healthcare utilization | Generated_Record_ID | Generated Record ID |
| SUS-ECDS | Healthcare utilization | Provider_Code | Provider code |
| SUS-ECDS | Healthcare utilization | EC_Department_Type | Department type |
| SUS-ECDS | Healthcare utilization | Der_Postcode_LSOA_2011_Code | LSOA code |
| SUS-ECDS | Healthcare utilization | Der_Postcode_CCG_Code | CCG code |
| SUS-ECDS | Healthcare utilization | GP_Practice_Code | GP practice code |
| SUS-ECDS | Healthcare utilization | Accommodation_Status_SNOMED_CT | Accommodation status (SNOMED CT) |
| SUS-ECDS | Healthcare utilization | EC_AttendanceCategory | Attendance category (SNOMED CT) |
| SUS-ECDS | Healthcare utilization | EC_Arrival_Mode_SNOMED_CT | Arrival mode (SNOMED CT) |
| SUS-ECDS | Healthcare utilization | EC_Attendance_Number | Attendance number (SNOMED CT) |
| SUS-ECDS | Healthcare utilization | Discharge_Destination_SNOMED_CT | Discharge destination (SNOMED CT) |
| SUS-ECDS | Healthcare utilization | Discharge_Follow_Up_SNOMED_CT | Discharge follow up (SNOMED CT) |
| SUS-ECDS | Healthcare utilization | EC_Acuity_SNOMED_CT | Acuity (SNOMED CT) |
| SUS-ECDS | Healthcare utilization | EC_Attendance_Source_SNOMED_CT | Attendance source (SNOMED CT) |
| SUS-ECDS | Healthcare utilization | EC_Discharge_Status_SNOMED_CT | Discharge status (SNOMED CT) |
| SUS-ECDS | Complications/adverse events | EC_Chief_Complaint_SNOMED_CT | Chief Complaint (SNOMED CT) |
| SUS-ECDS | Complications/adverse events | EC_Diagnosis_01 | Primary diagnosis (SNOMED CT) |
| SUS-ECDS | Healthcare utilization | EC_Treatment_01 | Primary treatment code (SNOMED CT) |
| SUS-ECDS | Complications/adverse events | Der_EC_Diagnosis_All | All diagnosis codes (SNOMED CT) |
| SUS-ECDS | Resident needs | Der_EC_Treatment_All | All treatment codes (SNOMED CT) |
| SUS-ECDS | Complications/adverse events | EC_Injury_Date | Injury date |
| SUS-ECDS | Complications/adverse events | EC_Injury_Time | Injury time |
| SUS-ECDS | Complications/adverse events | EC_Injury_Activity_Status_SNOMED | Injury activity status (SNOMED CT) |
| SUS-ECDS | Complications/adverse events | EC_Injury_Activity_Type_SNOMED_C | Injury activity type (SNOMED CT) |
| SUS-ECDS | Complications/adverse events | EC_Injury_Intent_SNOMED_CT | Injury intent (SNOMED CT) |
| SUS-ECDS | Complications/adverse events | EC_Injury_Mechanism_SNOMED_CT | Injury mechanism (SNOMED CT) |
| SUS-ECDS | Complications/adverse events | EC_Place_Of_Injury_SNOMED_CT | Place of injury (SNOMED CT) |
| SUS-ECDS | Complications/adverse events | AEA_Diagnosis_01 | Primary diagnosis code (AEA code) |
| SUS-ECDS | Healthcare utilization | AEA_Treatment_01 | Primary treatment code (AEA code) |
| SUS-ECDS | Complications/adverse events | Der_AEA_Diagnosis_All | All diagnosis codes (AEA codes) |
| SUS-ECDS | Resident needs | Der_AEA_Treatment_All | All treatment codes (AEA codes) |
| SUS-ECDS | Healthcare utilization | Org_Code_Patient_Pathway_ID_Issuer | Organisation code of the organisation that assigned the patient pathway identifier |
| SUS-ECDS | Demographics/characteristics | Month_of_Birth | Month of birth |
| SUS-ECDS | Demographics/characteristics | Year_of_Birth | Year of birth |
| SUS-ECDS | Demographics/characteristics | Sex | Sex |
| SUS-ECDS | Demographics/characteristics | Ethnic_Category | Ethnicity |
| SUS-OP | Healthcare utilization | Appointment_Date | Appointment date |
| SUS-OP | Healthcare utilization | Appointment_Time | Appointment time |
| SUS-OP | Healthcare utilization | Der_Activity_Month | Activity month |
| SUS-OP | Healthcare utilization | Der_Financial_Year | Financial year |
| SUS-OP | Healthcare utilization | Der_Provider_Code | Provider code |
| SUS-OP | Healthcare utilization | Der_Postcode_LSOA_Code | LSOA code |
| SUS-OP | Healthcare utilization | Der_Postcode_CCG_Code | CCG code |
| SUS-OP | Healthcare utilization | GP_Practice_Code | GP practice code |
| SUS-OP | Healthcare utilization | Der_Appointment_Type | Appointment type |
| SUS-OP | Healthcare utilization | Der_Attendance_Type | Attendance type |
| SUS-OP | Healthcare utilization | Attendance_Status | Attendance status |
| SUS-OP | Healthcare utilization | OPA_Referral_Source | Source of referral for the outpatient episode |
| SUS-OP | Healthcare utilization | Treatment_Function_Code | Treatment function code |
| SUS-OP | Healthcare utilization | Main_Speciality_Code | Main speciality code |
| SUS-OP | Resident needs | Der_Procedure_All | All procedure codes |
| SUS-OP | Demographics/characteristics | Month_of_Birth_SUS | Month of birth |
| SUS-OP | Demographics/characteristics | Year_of_Birth_SUS | Year of birth |
| SUS-OP | Demographics/characteristics | Sex | Sex |
| SUS-OP | Demographics/characteristics | Ethnic_Category | Ethnicity |
| PDS | Demographics/characteristics | Der_Practice_Code | GP practice code |
| PDS | Demographics/characteristics | Der_CCGofResidence | CCG code for residence location |
| PDS | Demographics/characteristics | Gender | Sex |
| PDS | Demographics/characteristics | DateofDeath | Date of death |
| PDS | Demographics/characteristics | Der_Postcode_LSOA_code | LSOA of residence |
| PDS | Demographics/characteristics | Der_DOBYearMnth | Year and month of birth |
| PDS | Demographics/characteristics | Change_Time_Stamp | Date and time to indicate when record was updated (for any column) |
| Care home residency^1^ and CQC dataset | Linkage | Der_CQC_Location | Pseudonymised CQC location id |
| Care home residency | Care home stay | Der_Start_Date | Start of care home stay |
| Care home residency | Care home stay | Der_End_Date | End of care home stay |
| Care home residency | Care home characteristics and workforce characteristics | Der_CQC_Service_Type | Type of care home |
| Care home residency | Demographics/characteristics | CCG_Of_Residence | CCG of care home |
| Care home residency | Care home characteristics and workforce characteristics | CareHomeIndicator | ? Unclear |
| Care home residency | Care home stay | DeathFlag | Flag to indicate resident died |
| Care home residency | Care home stay | YearMonthDeath_YYYYMM | Year and month of death |
| Care home residency | Care home stay | DeathInHospFlag | Flag to indicate death in hospital |
| Care home residency | Care home stay | LastResidencyFlag | Flag to indicate last residence of the person |
| Ambulance | Healthcare utilization | call_date | Date of call |
| Ambulance | Healthcare utilization | call_origin | Codes to identify the origin of the call into the ambulance control room, whether 999 was directly dialled or it was transferred from another agency. |
| Ambulance | Healthcare utilization | stop_codes | Codes to identify the reason the call was closed if no face to face response was received from the ambulance service. |
| Ambulance | Healthcare utilization | time_call_connected | Time call connected |
| Ambulance | Healthcare utilization | time_call_answered | Time call answered |
| Ambulance | Healthcare utilization | treatment_type | Codes to identify the overall outcome of the call. |
| Ambulance | Healthcare utilization | chief_complaint_call_triage_code | Codes to identify the initial chief complaint of the patient based on the information provided during telephone call to the ambulance control room. |
| Ambulance | Healthcare utilization | orgid_prov3 | Provider code 3 characters |
| Ambulance | Healthcare utilization | time_resource_arrived_on_scene | Time resource arrived on scene |
| Ambulance | Healthcare utilization | receiving_location_type_cad | ? Unclear |
| ASC CLD | Care home stay | Accommodation_Status_ASC | Accommodation status |
| ASC CLD | Care home stay | Client_Funding_Status_ASC | Client funding status |
| ASC CLD | Care home stay | Service_Component_ASC | Service component |
| ASC CLD | Care home stay | Service_Type_ASC | Service type |
| CSDS (CYP001MPI) | Demographics/characteristics | Gender | Person stated gender code or Sex |
| CSDS (CYP001MPI) | Demographics/characteristics | EthnicCategory | Ethnic category |
| CSDS (all files) | Healthcare utilization | EFFECTIVE_FROM | Date and time to indicate when record was updated (for any column) |
| CSDS (all files) | Healthcare utilization | RecordNumber | Record number |
| CSDS (CYP001MPI) | Healthcare utilization | LSOA | LSOA code |
| CSDS (CYP001MPI) | Healthcare utilization | Der_Postcode_yr2011_LSOA | LSOA code |
| CSDS (CYP001MPI) | Demographics/characteristics | AgeYr_RP_StartDate | Age of patient at reporting period start (days) |
| CSDS (CYP001MPI) | Care home stay | AgeYr_Death | Age at death (years) |
| CSDS (CYP001MPI) | Care home stay | Age_Death | Age at death (days) |
| CSDS (CYP001MPI) | Healthcare utilization | OrgIDICBRes | Organisation identier (ICB of residence) |
| CSDS (CYP001MPI) | Palliative care needs | DiscussPreferredDeathLocation_Indicator | Preferred death location discussed indicator |
| CSDS (CYP001MPI) | Palliative care needs | DeathLocationPreferred_Type | Death location type code (preferred) |
| CSDS (CYP001MPI) | Palliative care needs | DeathLocationActual_Type | Death location type code (actual) |
| CSDS (CYP001MPI) | Palliative care needs | NotAtPreferredLocation_Reason | Death not at preferred location reason |
| CSDS (CYP201CareContact) | Healthcare utilization | CareContactID | Care contact identifier |
| CSDS (CYP201CareContact) | Healthcare utilization | ServiceRequestID | Service request identifier |
| CSDS (CYP201CareContact) | Healthcare utilization | Contact_Date | Care contact date |
| CSDS (CYP201CareContact) | Healthcare utilization | Consultation_Type | Consultation type |
| CSDS (CYP201CareContact) | Healthcare utilization | Consultation_MediumUsed | Consultation mechanism e.g. face to face |
| CSDS (CYP201CareContact) | Healthcare utilization | Activity_LocationType | Activity location type code |
| CSDS (CYP201CareContact) | Healthcare utilization | OrgID_Provider | Organisation identifier (code of provider) |
| CSDS (CYP201CareContact) | Healthcare utilization | AgeYr_Contact_Date | Age at care contact date |
| CSDS (CYP201CareContact) | Healthcare utilization | AttendanceStatus | Attendance status |
| CSDS (CYP101Referral) | Healthcare utilization | SourceOfReferral | Source of referral |
| CSDS (CYP101Referral) | Healthcare utilization | Referring_StaffGroup | Referring care professional staff group (community care) |
| CSDS (CYP101Referral) | Healthcare utilization | PrimaryReferralReason | Primary reason for referral (community care) |
| CSDS (CYP102ServiceTypeReferredTo) | Healthcare utilization | ServiceRequestID | Service request identifier |
| CSDS (CYP102ServiceTypeReferredTo) | Healthcare utilization | TeamID_Local | Care professional team local identifier |
| CSDS (CYP102ServiceTypeReferredTo) | Healthcare utilization | TeamType | Service or team type referred to (community care) |
| CQC | Care home characteristics and workforce characteristics | Location_HSCA_start_date | Start of registration at location |
| CQC | Care home characteristics and workforce characteristics | Care_home? | Care home or not |
| CQC | Care home characteristics and workforce characteristics | Care_homes_beds | Number of beds |
| CQC | Care home characteristics and workforce characteristics | Location_Inspection_Directorate | Inspection Directorate |
| CQC | Care home characteristics and workforce characteristics | Location_Primary_Inspection_Cate | Primary inspection category |
| CQC | Care home characteristics and workforce characteristics | Publication_Date | Publication date |
| CQC | Care home characteristics and workforce characteristics | Location_Region | Region |
| CQC | Care home characteristics and workforce characteristics | Location_NHS_Region | NHS Region |
| CQC | Care home characteristics and workforce characteristics | Location_Local_Authority | Local Authority |
| CQC | Care home characteristics and workforce characteristics | Location_ONSPD_CCG | CCG |
| CQC | Care home characteristics and workforce characteristics | service_type | Service type e.g. nursing home |
| CQC | Care home characteristics and workforce characteristics | Location_Type | Location type |
| CQC | Care home characteristics and workforce characteristics | Rating_overall | Rating: overall |
| CQC | Care home characteristics and workforce characteristics | Rating_caring | Rating: caring |
| CQC | Care home characteristics and workforce characteristics | Rating_well_led | Rating: well led |
| CQC | Care home characteristics and workforce characteristics | Rating_effective | Rating: effective |
| CQC | Care home characteristics and workforce characteristics | Rating_reponsive | Rating: responsive |
| CQC | Care home characteristics and workforce characteristics | Rating_safe | Rating: safe |
| CQC | Care home characteristics and workforce characteristics | serv_user_dementia | Service users with dementia |
| CQC | Care home characteristics and workforce characteristics | years_since_registration | Years since registration |

^1^Dataset abbreviations: ASC-CLD = Adult Social Care Client Level Dataset; CQC = Care Quality Commission; CSDS = Community Services Data Set; PDS = Personal Demographics Service; SUS-APC = Secondary Uses Service Admitted Patient Care; SUS-ECDS = Secondary Uses Service Emergency Care Dataset; SUS-OP = Secondary Uses Service Outpatient.

**APPENDIX 6 – Completion of measures added into care home digital care record software, by wave, for linked DCR data***

| **Measure** | **N** | **i. Complete both** | **ii. Wave 1 only** | **iii. Wave 2 only** | **iv. Missing both ¹** | **Of ii…**  **Resident died before Wave 2** | **Of ii… care home drop out or non-complete**¹ |
| --- | --- | --- | --- | --- | --- | --- | --- |
| MDS CPS | 767 | 58.7% | 21.1% | 2.4% | 17.8% | 27.8% | 45.7% |
| Barthel | 767 | 35.2% | 38.6% | 1.6% | 24.6% | 16.2% | § 78.7% |
| IAGeD | 767 | 55.3% | 20.6% | 4.0% | 20.1% | 26.6% | 46.8% |
| ASCOT-Proxy-Resident | 767 | 44.7% | 18.9% | 4.0% | 32.3% | 27.6% | 46.2% |
| ASCOT: Anxiety/low mood | 767 | 44.9% | 18.9% | 6.5% | 29.7% | 31.7% | 51.0% |
| ASCOT: Pain | 767 | 51.4% | 20.7% | 2.6% | 25.3% | 29.6% | 49.1% |
| ICECAP-O | 767 | 36.6% | 37.6% | 1.3% | 24.5% | 18.8% | §§ 76.0% |
| EQ-5D-5L Proxy | 767 | 60.5% | 21.8% | 2.4% | 15.4% | 28.1% | 44.9% |
| ASCS QoL item | 767 | 56.6% | 21.1% | 2.2% | 20.1% | 29.0% | 48.8% |

*Since data on resident death between waves or grouping by care home were only available once the DCR data had been linked to other data, we only consider those residents who were eligible for data linkage, but do not omit residents with data at Wave 2 only (see Figure 1, ).

¹ Due to drop out of n=5 care homes from Wave 1 to Wave 2, except when noted below (under § and §§).

§ Eleven additional care homes (**Provider 2 only**) returned no Barthel data at Wave 2 (n=157 (53.0%)). No additional information given as to why.

§§ Five additional care homes (**Provider 2 only**) returned no ICECAP-O data at Wave 2, although care staff completed other measures (n=96 (33.3%)). Despite a request for additional information, no reason was given for this omission.

**APPENDIX 7: GP data items possible to access from one ICS**

| **Type** | **Variable** |
| --- | --- |
| Demographics | Ethnicity |
| Demographics | BMI |
| Demographics | Age in Years |
| Demographics | Has died Y/N /month and year of death |
| Demographics | Care home flag in GP record and/ or ICS system |
| Demographics | Registration with GP practice aligned to care home declined |
| Risk stratification | End-of-life pathway register |
| Risk stratification | Place of death discussed |
| Risk stratification | Preferred place of death |
| Risk stratification | Infection - urinary tract |
| Risk stratification | Infection - chest - lower respiratory tract infection |
| Risk stratification | Infection - skin |
| Risk stratification | Injury resulting from fall |
| Risk stratification | Haematological malignancies |
| Risk stratification | Depression |
| Risk stratification | Dementia |
| Risk stratification | Incontinence - fecal |
| Risk stratification | Delirium |
| Risk stratification | Difficulty swallowing |
| Risk stratification | Frailty index (eFi) |
| Risk stratification | eFI: activity limitation |
| Risk stratification | eFI: anaemia and haematinic deficiency |
| Risk stratification | eFI: arthritis |
| Risk stratification | eFI: atrial fibrillation |
| Risk stratification | eFI: cerebrovascular disease |
| Risk stratification | eFI: chronic kidney disease (CKD) |
| Risk stratification | eFI: diabetes |
| Risk stratification | eFI: dizziness |
| Risk stratification | eFI: dyspnoea |
| Risk stratification | eFI: falls |
| Risk stratification | eFI: foot problems |
| Risk stratification | eFI: fragility fracture |
| Risk stratification | eFI: hearing impairment |
| Risk stratification | eFI: heart failure |
| Risk stratification | eFI: heart valve disease |
| Risk stratification | eFI: housebound |
| Risk stratification | eFI: hypertension |
| Risk stratification | eFI: hypotension/syncope |
| Risk stratification | eFI Memory & cognitive problems |
| Risk stratification | eFI Mobility and transfer problems |
| Risk stratification | eFI: osteoporosis |
| Risk stratification | eFI: Parkinsonism and tremor |
| Risk stratification | eFI: peptic ulcer |
| Risk stratification | eFI Peripheral vascular disease |
| Risk stratification | eFI: polypharmacy |
| Risk stratification | eFI: requirement for care |
| Risk stratification | eFI: respiratory disease |
| Risk stratification | eFI: skin ulcer |
| Risk stratification | eFI: sleep disturbance |
| Risk stratification | eFI: social vulnerability |
| Risk stratification | eFI: thyroid disease |
| Risk stratification | eFI: urinary incontinence |
| Risk stratification | eFI: urinary system disease |
| Risk stratification | eFI: visual impairment |
| Risk stratification | eFI: weight loss and anorexia |
| Risk stratification | eFI: ischaemic heart disease |
| Medications | Numbers of current prescriptions |
| Medications | Most recent medication review |
| Medications | Penicillins (BFN 5.1.1) |
| Medications | Cephalosporins, carbapenems & other beta-lactams (BNF 5.1.2) |
| Medications | Tetracyclines (BNF 5.1.3) |
| Medications | Aminoglycosides (BNF 5.1.4) |
| Medications | Macrolides (BNF 5.1.5) |
| Medications | Sulfonamides and trimethoprim (BNF 5.1.8) |
| Medications | Quinolones (BNF 5.1.12) |
| Medications | Non-opioid analgesics (BNF 4.7.1) |
| Medications | Compound analgesic preparations (BNF 4.7.1) |
| Medications | Opioid analgesics (BNF 4.7.2) |
| Medications | Non-steroidal anti-inflammatory drugs (BNF 10.1.1) |
| Medications | Other drugs used as analgesics |
| Vaccinations | Flu |
| Vaccinations | Covid 19 |
| Vaccinations | Pneumonia |
| Appointments/utilisation | Number of appointments by staff type |
| Appointments/utilisation | EHCH MDT referral |
| Appointments/utilisation | Hospital referral recorded? |
| Appointments/utilisation | A&E referral recorded? |
| Appointments/utilisation | Continence service referral recorded? |
| Appointments/utilisation | Community services referral recorded? |
| Appointments/utilisation | Community nurse referral recorded |
| Appointments/utilisation | Referrals to falls service |
| Appointments/utilisation | Face to Face |
| Appointments/utilisation | Telephone appointments |

**APPENDIX 8 – Reason for non-inclusion of planned variables in final MDS**

| **Domain** | **Variable** | **Expected source^1^** | **Reason for not included/ reason for different source** |
| --- | --- | --- | --- |
| Demographics/ characteristics | Date of birth  Sex registered at birth  Religion  First language  Marital status  Ethnicity  Weight  Height  Area-based deprivation (last known residence) | PDS  PDS  DCRs  DCRs  DCRs  DCRs  DCRs, GP data  DCRs, GP data  PDS linked to public IMD data | Included, but sourced from PDS and SUS  Included, but sourced from PDS, SUS, CSDS  Included, but high % missing data  Included, but high % missing data  Included, but high % missing data  Included, but high % missing data  Included, but sourced from DCRs only  Included, but sourced from DCRs only  The version of PDS we accessed did not allow access to location of previous address |
| Palliative care needs | End-of-life pathway register | GP data | No access to GP data, but sourced information from CSDS |
| Care home stay | Date of death  Admitted from hospital or community | PDS  SUS | Included, but sourced from PDS and SUS  Included, but limited to discharge from hospital to care home in previous year |
| Resident needs | Visual impairment  Hearing impairment  Cognitive impairment  Oral and nutritional status  Continence | GP data  GP data  DCRs, GP data, SUS  GP data, SUS  DSRs, GP data SUS | No access to GP data  No access to GP data  Included, but sourced from DCRs and SUS only  Included, but sourced from SUS only  Included, but sourced from DCRs and SUS only |
| Quality of life |  |  |  |
| Complications/ adverse events | Infections  Falls leading to GP or hospital visit  Falls (recorded in care home only)  NEWS2/RESTORE | DCRs, GP data, SUS  DCRs, GP data, SUS, 999 / ambulance data  DCRs  DCRs | Included, but sourced from SUS only and limited to upper and lower respiratory tract infections. Not able to extract from DCRs in standardised format.  Included, but sourced from SUS only. Field in ambulance dataset required to derive this was 100% missing. Not able to extract from DCRs in standardised format.  Not able to extract in standardised format.  Included, but 100% missing |
| Diagnoses | Medical history  Frailty  Adverse reactions and allergies | GP data, SUS  GP data, SUS  GP data | Included, but sourced from SUS only  Included, but sourced from SUS only  No access to GP data |
| Medications/ vaccinations | Prescribed medications | GP data | No access to GP data |
|  | Administered vaccinations |  |  |
| Healthcare utilisation | Primary care use  Out of hours contacts  Ambulance attendances for falls | GP data, NHS111, ambulance  GP data  Ambulance | Included, but sourced from ambulance data only.  No access to GP data. However, we derived out of hours ambulance callouts.  Field in dataset required to derive this was 100% missing so could not use to get reason for attendances |
|  | Ambulance conveyances to ED |  | Field in dataset required to derive this was populated differently to as expected from data specifications |

^1^ Dataset abbreviations: CSDS = Community Services Data Set; DCR = Digital Care Record; PDS = Personal Demographics Service; SUS = Secondary Uses Service

**APPENDIX 9 - Comparison of variables with inconsistent definitions across data sources**

A small number of comorbidities were recorded in both hospital and care home records. However, these variables were not defined in a consistent way. Even within a data source, there can be several established definitions and code lists, which will give slightly different results. Table a shows that 11% of residents in our sample are identified as having dementia from SUS data according to one definition but not another (53 + 13/583).

The comorbidities were also collected differently: variables in the care home data were recorded at one particular point in time - therefore reflecting the resident’s health status at that moment. For the SUS data, we collected information relating to these conditions over a three-year look-back period. For acute conditions, there were high levels of discrepancy between SUS and care home DCRs. For example, delirium was recorded in hospital records for 145 patients in the previous 3 years, but only 20 (14%) of these were recorded as delirious by care home staff at the time of recording. However, there were also 30 (48%, 30/62) residents recorded with delirium in the care home DCRs that were not reflected in hospital records prior to that date. For cognitive impairment, which tends to not improve over time, there was more consistency, although still substantial disagreement. Agreement between care home DCR and SUS record of dementia is recorded in Table 3 in main results.

Table a) Comparison of residents identified to have dementia based on Charlson index code list and Frailty syndromes code list

| Comparing dementia prevalence within SUS | | Frailty syndromes code list | | | |
| --- | --- | --- | --- | --- | --- |
|  |  | No | Yes | Missing | Total |
| Charlson index code list | No | 189 | 13 | 0 | 202 |
|  | Yes | 53 | 328 | 0 | 381 |
|  | Missing | 0 | 0 | 144 | 144 |
|  | Total | 242 | 341 | 144 | 727 |

Table b) Comparison of residents identified to have delirium based on SUS data using Soong et al. List of frailty syndromes and care home DCR using I-AGED

| Delirium | | Care home DCR | | | |
| --- | --- | --- | --- | --- | --- |
|  |  | No | Yes | Missing | Total |
| SUS | No | 270 | 30 | 73 | 373 |
|  | Yes | 145 | 20 | 45 | 210 |
|  | Missing | 105 | 12 | 27 | 144 |
|  | Total | 520 | 62 | 145 | 727 |

Table c) Comparison of residents identified to have cognitive impairment based on Soong et al. list of frailty syndromes and care home DCR, assess using Morris et al.

| Cognitive impairment | | Care home DCR | | | | | | | | | | | | |
| --- | --- | --- | --- | --- | --- | --- | --- | --- | --- | --- | --- | --- | --- | --- |
|  |  | Intact | Borderline intact | Mild impair-ment | | Moderate impair-ment | Moderately severe impairment | Sever impair-ment | Very severe impair-ment | | Missing | | Total | |
| SUS | No | 13 | 52 | 13 | 10 | | 8 | 7 | 6 | 17 | | 126 | |  |
|  | Yes | 33 | 45 | 61 | 84 | | 52 | 60 | 43 | 79 | | 457 | |  |
|  | Missing | 10 | 19 | 11 | 17 | | 20 | 21 | 27 | 19 | | 144 | |  |
|  | Total | 56 | 116 | 85 | 111 | | 80 | 88 | 76 | 115 | | 727 | |  |

Note: 144 people are missing for each of the SUS measures as no inpatient hospital record

**APPENDIX 10 – Complete final prototype MDS.**

| **Domain** | **Variable** | **Categories (if categorical)** | **n^1^** | **Mean (SD) or %** |
| --- | --- | --- | --- | --- |
| **Demographics/ characteristics** | Ethnicity (final)^2^ | White | 692 | 95% |
|  |  | Black or Black British | <=5 | NA |
|  |  | Asian or Asian British | <=5 | NA |
|  |  | Mixed | <=5 | NA |
|  |  | Other | <=5 | NA |
|  |  | Missing | 25 | 3% |
|  | Sex (final)^2^ | Female | 513 | 71% |
|  |  | Male | 214 | 29% |
|  | Date of birth record (final)^2^ | Available | >=720 | 99% |
|  |  | Missing | <=5 | NA |
|  | Date of death present in record (final)^2^ | Present | 58 | 8% |
|  |  | Not present | 669 | 92% |
|  | Religion | Christianity | 93 | 13% |
|  |  | Buddhist | <=5 | NA |
|  |  | Other | 8 | 1% |
|  |  | No religion | 13 | 2% |
|  |  | Missing | >=610 | 84% |
|  | Marital status | Divorced/separated/single | 15 | 2% |
|  |  | Married/cohabiting | 40 | 6% |
|  |  | Widowed | 54 | 7% |
|  |  | Missing | 618 | 85% |
|  | First language spoken | English | 160 | 22% |
|  |  | Other | <=5 | NA |
|  |  | Missing | >=561 | 77% |
|  | Power of attorney | Yes | 61 | 8% |
|  |  | No | 103 | 14% |
|  |  | Missing | 563 | 77% |
|  | Deprivation of Liberty status | Yes | 126 | 17% |
|  |  | No | 544 | 75% |
|  |  | Missing | 57 | 8% |
|  | DNACPR status | Yes | 572 | 79% |
|  |  | No | >=150 | 21% |
|  |  | Missing | <=5 | NA |
|  | Weight | 20-35kg | 7 | 1% |
|  |  | 36-50kg | 109 | 15% |
|  |  | 51-65kg | 232 | 32% |
|  |  | 66-80kg | 152 | 21% |
|  |  | 81-95kg | 43 | 6% |
|  |  | 96-110kg | 21 | 3% |
|  |  | 111-125kg | <=5 | NA |
|  |  | 126-140kg | <=5 | NA |
|  |  | Missing | 158 | 22% |
|  | Height | 111-125cm | 8 | 1% |
|  |  | 126-150cm | 67 | 9% |
|  |  | 151-170cm | 508 | 70% |
|  |  | 171-190cm | 130 | 18% |
|  |  | 191-210cm | <=5 | NA |
|  |  | Missing | >=8 | 1% |
| **Palliative care needs** | Discussed preferred death location indicator | Yes | 18 | 3% |
|  |  | No | 383 | 53% |
|  |  | Missing | 326 | 45% |
|  | Preferred death location | Care home | 7 | 1% |
|  |  | Care home services with nursing | 27 | 4% |
|  |  | Care home services without nursing | 51 | 7% |
|  |  | Hospice | <=5 | NA |
|  |  | Hospital | <=5 | NA |
|  |  | Patient's own home | 16 | 2% |
|  |  | Other (not listed) | <=5 | NA |
|  |  | Missing | 623 | 86% |
| **Care home stay** | Client funding status | Health funded | 7 | 1% |
|  |  | Social care funded | 18 | 2% |
|  |  | Client funded | 19 | 3% |
|  |  | Joint client and social care funded | 96 | 13% |
|  |  | Other | <=5 | NA |
|  |  | Unknown in record | 77 | 11% |
|  |  | Missing | >=505 | 70% |
|  | Discharge from an in-patient spell to a care home (1 year history) | Yes | 32 | 4.40% |
|  |  | No | 695 | 95.5% |
|  | Death in hospital in the period between the index date and end of study | Yes | <=5 | NA |
|  |  | No | >=720 | 99% |
|  | Length of stay in care home |  |  | 876.94 (812.60) |
| **Resident needs** | Cognitive impairment | Borderline intact | 56 | 8% |
|  |  | Intact | 116 | 16% |
|  |  | Mild impairment | 85 | 12% |
|  |  | Moderate impairment | 111 | 15% |
|  |  | Moderately severe impairment | 80 | 11% |
|  |  | Severe impairment | 88 | 12% |
|  |  | Very severe impairment | 76 | 10% |
|  |  | Missing | 115 | 16% |
|  | Bowel continence | Continent | 201 | 28% |
|  |  | Incontinent | 289 | 40% |
|  |  | Occasional accident | 109 | 15% |
|  |  | Missing | 128 | 18% |
|  | Bladder continence | Continent | 143 | 20% |
|  |  | Incontinent/Catheter | 329 | 45% |
|  |  | Occasional accident | 124 | 17% |
|  |  | Missing | 131 | 18% |
|  | ASCOT: Pain | High-level needs | 21 | 3% |
|  |  | Some needs | 59 | 8% |
|  |  | No needs | 263 | 36% |
|  |  | Ideal state | 210 | 29% |
|  |  | Missing | 174 | 24% |
|  | ASCOT: Anxiety and low mood |  |  | 3.97 (1.45) |
|  | Food texture requirements | IDDSI 3 - Liquidised | <=5 | NA |
|  |  | IDDSI 4 - Pureed | 41 | 6% |
|  |  | IDDSI 5 - Minced & Moist | 47 | 6% |
|  |  | IDDSI 6 - Soft & Bite-sized | 65 | 9% |
|  |  | IDDSI 7 - Easy to Chew | 34 | 5% |
|  |  | IDDSI 7 - Regular | 482 | 66% |
|  |  | Missing | >=54 | 8% |
|  | Drink thickness requirements | IDDSI 0 - Thin | 598 | 82% |
|  |  | IDDSI 1 - Slightly thick | 30 | 4% |
|  |  | IDDSI 2 - Mildly thick | 23 | 3% |
|  |  | IDDSI 3 - Moderately thick | <=5 | NA |
|  |  | IDDSI 4 - Extremely thick | <=5 | NA |
|  |  | Missing | 71 | 10% |
|  | Food allergy | Yes | 26 | 4% |
|  |  | No | 633 | 87% |
|  |  | Missing | 68 | 9% |
|  | Contact allergy | Yes | <=5 | NA |
|  |  | No | 156 | 21% |
|  |  | Missing | >=564 | 78% |
|  | Medication allergy | Yes | 73 | 10% |
|  |  | No | 88 | 12% |
|  |  | Missing | 566 | 78% |
|  | Penicillin allergy | Yes | 27 | 4% |
|  |  | No | 134 | 18% |
|  |  | Missing | 566 | 78% |
|  | Pressure ulcers (Waterlow score) (reported for 527 residents/ 28% missing) | |  | 17.87 (7.30) |
|  | Pressure ulcers (Braden score) (reported for 338 residents/ 54% missing) | |  | 16.61 (4.02) |
|  | Delirium (I-AGED score) (reported for 582 residents/ 20% missing) | |  | 1.11 (1.78) |
|  | Functional independence (Barthel score) (reported for 566 residents/ 22% missing) | |  | 41.40 (30.26) |
|  | Number of ED attendance or outpatient appointments with nasogastric feeding procedure | |  | NA |
| **Quality of life** | Quality of Life overall (reported for 596 residents/ 18% missing) | So good, it could not be better | 19 | 3% |
|  |  | Very good | 157 | 22% |
|  |  | Good | 201 | 28% |
|  |  | Alright | 171 | 24% |
|  |  | Bad | 32 | 4% |
|  |  | Very bad | 9 | 1% |
|  |  | So bad, it could not be worse | 7 | 1% |
|  |  | Missing | 131 | 18% |
|  | Ascot Proxy-Resident (reported for 488 residents/ 33% missing) | |  | 0.83 (0.19) |
|  | ICECAP-O (reported for 569 residents/ 22% missing) | |  | 0.73 (0.21) |
|  | UK Crosswalk (reported for 631 residents/ 13% missing) | |  | 0.33 (0.35) |
| **Complications/ adverse events** | MUST (malnutrition universal scoring tool) score | 0 | 101 | 14% |
|  |  | 1 | 23 | 3% |
|  |  | 2 | 20 | 3% |
|  |  | 3 | 12 | 2% |
|  |  | 4 | 6 | 1% |
|  |  | 5 | <=5 | NA |
|  |  | Missing | >=562 | 78% |
|  | Frequency of in-patient admissions with upper respiratory tract infection recorded (1 year history) | |  | 0.01 (0.10) |
|  | Frequency of in-patient admissions with lower respiratory tract infection recorded (1 year history) | |  | NA |
| **Diagnoses**  (based on previous 3 years hospital admission diagnosis codes)  (reported for 583 residents/ 20% missing) apart from ‘dementia (final)’ | Dementia (final)^2^ | | 514 | 71% |
|  | *Elixhauser conditions^3^* | |  |  |
|  | Number of Elixhauser conditions | |  | 3.59 (2.34) |
|  | 2 or more Elixhauser conditions | | 470 | 81% |
|  | Alcohol abuse | | 22 | 4% |
|  | Anaemia | | 83 | 14% |
|  | Cardiac arrhythmias | | 189 | 32% |
|  | Chronic pulmonary disease | | 110 | 19% |
|  | Coagulopathy | | 15 | 3% |
|  | Congestive heart failure | | 86 | 15% |
|  | Depression | | 129 | 22% |
|  | Diabetes (complicated and uncomplicated) | | 127 | 22% |
|  | Drug abuse | | <=5 | NA |
|  | Fluid and electrolyte disorders | | 226 | 39% |
|  | Hemiplegia / paraplegia | | 20 | 3% |
|  | Hypertension (complicated and uncomplicated) | | 353 | 61% |
|  | Hypothyroidism | | 75 | 13% |
|  | Liver disease | | 30 | 5% |
|  | Lymphoma | | 6 | 1% |
|  | Metastatic solid tumour / metastatic cancer | | 11 | 2% |
|  | Obesity | | 39 | 7% |
|  | Other neurological disorders | | 154 | 26% |
|  | Peptic ulcer disease excl bleeding | | 6 | 1% |
|  | Psychoses | | 13 | 2% |
|  | Pulmonary circulation disorders | | 25 | 4% |
|  | Peripheral vascular disease | | 47 | 8% |
|  | Rheumatoid arthritis / collagen vascular diseases | | 179 | 31% |
|  | Renal failure | | 39 | 7% |
|  | Solid tumour without metastasis | | 22 | 4% |
|  | Valvular disease | | 67 | 11% |
|  | Weight loss | | 19 | 3% |
|  | *Frailty syndromes^4^* | |  |  |
|  | Number of frailty syndromes | |  | 2.17 (1.81) |
|  | Cognitive impairment (delirium, dementia, senility) | | 457 | 78% |
|  | Anxiety/Depression | | 168 | 29% |
|  | Functional dependence | | 102 | 17% |
|  | Falls/Fractures | | 291 | 50% |
|  | Incontinence | | 105 | 18% |
|  | Mobility problems | | 217 | 37% |
|  | Pressure ulcers | | 62 | 11% |
| **Healthcare utilisation** |  | | *n (people with at least one event)* | *% who had at least one event* |
|  | Elective admissions (1 year history) | | 65 | 9% |
|  | Emergency admissions (1 year history) | | 284 | 39% |
|  | Potentially avoidable emergency admissions (1 year history) ^5^ | | 119 | 16% |
|  | Emergency department attendances (1 year history) | | 370 | 51% |
|  | Emergency department attendances via ambulance (1 year history) | | 331 | 46% |
|  | Emergency department attendances after 6pm/ before 8am (1 year history) | | 218 | 30% |
|  | Emergency department attendances lasting more than 12 hours (1 year history) | | 156 | 21% |
|  | Emergency department attendances for falls (1 year history) | | 18 | 2% |
|  | Outpatient appointments (1 year history) | | 236 | 32% |
|  | Missed outpatient appointments (1 year history) | | 28 | 4% |
|  | Community services appointments (1 year history) | | 608 | 84% |
|  | Speech and language therapy appointments (1 year history) | | 49 | 7% |
|  | Continence appointments (1 year history) | | 159 | 26% |
|  | District nursing appointments (1 year history) | | 398 | 55% |
|  | Podiatry appointments (1 year history) | | 31 | 4% |
|  | Community rehabilitation appointments (1 year history) | | 79 | 11% |
|  | Face to face community services appointments (1 year history) | | 444 | 61% |
|  | Missed community services appointments (1 year history) | | 22 | 3% |
|  | Ambulance call outs (1 June - 31 October 2023) | | 197 | 27% |
|  | Ambulance attendances (1 June - 31 October 2023) | | 195 | 27% |
|  | Ambulance attendances after 6pm/ before 8am (1 June - 31 October 2023) | | 118 | 16% |
|  | Ambulance conveyances (1 June - 31 October 2023) | | 147 | 20% |
|  |  | | *Total activity* | *Mean (SD)* |
|  | Average number of emergency admissions (1 year history) | | 451 | 0.62 (1.02) |
|  | Average number of elective admissions (1 year history) | | 97 | 0.13 (0.60) |
|  | Average number of potentially avoidable emergency admissions (1 year history) ^5^ | | 143 | 0.20 (0.48) |
|  | Average number of emergency department attendances (1 year history) | | 752 | 1.03 (1.49) |
|  | Average number of emergency department attendances after 6pm/ before 8am (1 year history) | | 331 | 0.46 (0.85) |
|  | Average number of emergency department attendances lasting more than 12 hours (1 year history) | | 206 | 0.28 (0.63) |
|  | Average number of emergency department attendances via ambulance (1 year history) | | 605 | 0.83 (1.27) |
|  | Average number of emergency department attendances due to a fall (1 year history) | | 21 | 0.03 (0.20) |
|  | Average number of outpatient appointments (1 year history) | | 424 | 0.58 (1.47) |
|  | Average number of missed outpatient appointments (1 year history) | | 29 | 0.04 (0.20) |
|  | Average number of community services appointments (1 year history) | | 15266 | 21.00 (65.83) |
|  | Average number of missed community services appointments (1 year history) | | 36 | 0.05 (0.33) |
|  | Average number of face to face community services appointments (1 year history) | | 6720 | 9.24 (30.47) |
|  | Average number of district nursing appointments (1 year history) | | 11347 | 15.61 (61.82) |
|  | Average number of speech and language therapy appointments (1 year history) | | 155 | 0.21 (1.03) |
|  | Average number of podiatry appointments (1 year history) | | 286 | 0.39 (3.98) |
|  | Average number of continence appointments (1 year history) | | 373 | 0.51 (1.44) |
|  | Average number of community rehabilitation appointments (1 year history) | | 470 | 0.65 (3.85) |
|  | Average number of ambulance call outs (1 June-31 October 2023) | | 333 | 0.45 (0.97) |
|  | Average number of ambulance attendances (1 June-31 October 2023) | | 325 | 1.65 (1.21) |
|  | Average number of ambulance attendances after 6pm/ before 8am (1 June-31 October 2023) | | 156 | 0.21 (0.57) |
|  | Average number of ambulance conveyances (1 June-31 October 2023) | | 210 | 0.29 (0.72) |
| **Care home characteristics and workforce characteristics** | Service type | Nursing | 403 | 55% |
|  |  | Nursing and Residential | 49 | 7% |
|  |  | Residential | 262 | 36% |
|  |  | Missing | 13 | 2% |
|  | Registered bed capacity | Less than 50 | 211 | 29% |
|  |  | 50 or more | 485 | 67% |
|  |  | Missing | 31 | 4% |
|  | CQC rating | Outstanding | 72 | 10% |
|  |  | Good | 511 | 70% |
|  |  | Requires improvement | 113 | 16% |
|  |  | Missing | 31 | 4% |
|  | Years of service registration | Less than 10 years | 238 | 33% |
|  |  | More than 10 years | 458 | 63% |
|  |  | Missing | 31 | 4% |
|  | Number of beds (reported for 696 residents/ 4% missing) | |  | 54.33(16.68) |
|  | Number of beds currently occupied (reported for 696 residents/ 4% missing) | |  | 42.54(14.75) |
|  | Number of resident fully self-funding (reported for 650 residents/ 11% missing) | |  | 22.41(16.26) |
|  | Number of staff (reported for 696 residents/ 4% missing) | |  | 67.52(32.16) |
|  | Number of full-time staff (reported for 696 residents/ 4% missing) | |  | 45.55(20.35) |
|  | Number of part-time staff (reported for 659 residents/ 9% missing) | |  | 19.32(13.42) |
|  | Number of staff on permanent contracts (reported for 659 residents/ 9% missing) | |  | 62.54(28.14) |
|  | Number of staff vacancies (reported for 696 residents/ 31% missing) | |  | 3.77(10.36) |
|  | Number of agency staff (reported for 600 residents/ 17% missing) | |  | 1.52(4.28) |
|  | Number of care workers (reported for 659 residents/ 9% missing) | |  | 32.44(20.69) |
|  | Number of senior care workers (reported for 659 residents/ 9% missing) | |  | 9.45(4.43) |
|  | Number of registered nurses (reported for 644 residents/ 11% missing) | |  | 5.05(6.07) |
|  | Number of nursing associates (reported for 632 residents/ 13% missing) | |  | 0.43(1.31) |
|  | Number of nursing assistants (reported for 632 residents/ 13% missing) | |  | 0.31(0.89) |
|  | Number of allied health professionals (reported for 632 residents/ 13% missing) | |  | 0(0) |
|  | Number of activities coordinators (reported for 659 residents/ 9% missing) | |  | 1.75(1.38) |
|  | Number of staff in roles above on permanent contracts (reported for 632 residents/ 13% missing) | |  | 43.98(25.68) |
|  | Number of staff vacancies in roles above (reported for 632 residents/ 13% missing) | |  | 1.93(1.84) |
|  | Number of agency staff in roles above (reported for 592 residents/ 19% missing) | |  | 0.71(3.67) |

^1^ Numbers are reported for 727 residents unless otherwise specified

^2^Reporting variable as created in the hierarchy process – see Table 3

^3^Elixhauser list of comorbidities (E Elixhauser A, Steiner C, Harris DR, et al. Comorbidity measures for use with administrative data. Med Care. 1998;36:8–27. doi: 10.1097/00005650-199801000-00004; Quan H, Sundararajan V, Halfon P, et al. Coding algorithms for defining comorbidities in ICD-9-CM and ICD-10 administrative data. Med Care. 2005;43:1130–9. doi: 10.1097/01.MLR.0000182534.19832.83)

^4^Frailty Syndromes (Soong J, Poots AJ, Scott S, et al. Developing and validating a risk prediction model for acute care based on frailty syndromes. BMJ Open. 2015;5. doi: 10.1136/BMJOPEN-2015-008457)

^5^Potentially avoidable emergency admissions (Care Quality Commission, Great Britain. Parliament. House of Commons. The state of health care and adult social care in England in 2012/13. 2013;86.).

**APPENDIX 11 – Using MDS data to understand ED attendance and ambulance activity for care home residents: a worked example**

In the main report, we presented key variables from the MDS with a full description of the MDS in Appendix 8. However, a key benefit of an MDS is the ability to explore sub groups of residents. These tables are examples of analyses that could help understand whether there are differences in outcomes or activities in different subgroups. Tables below are examples of such subgroup analyses which were suggested by stakeholders as of interest. For example, in our sample population, activity was in general higher across both A&E and ambulance services for those in residential care homes, compared to nursing homes, which could be informative when commissioning local services.

Table a) ED attendances for year leading to index date 1. * where no mean reported as denominator <=5. Mean reports the mean of all those eligible e.g. can only have ED attendance via ambulance if you have had an ED attendance

| **Variable** | **Total** | **Sex** | | **Age** | | | | **Nursing vs residential** | | | **Dementia** | | | **Deprivation^1^** | | | | | |
| --- | --- | --- | --- | --- | --- | --- | --- | --- | --- | --- | --- | --- | --- | --- | --- | --- | --- | --- | --- |
|  |  | **Female** | **Male** | **<65** | **65-79** | **>=80** | **Missing** | **Nursing** | **Residential** | **Missing** | **No** | **Yes** | **Missing** | **Most deprived fifth** | **Second most deprived quintile** | **Middle fifth** | **Second least deprived fifth** | **Least deprived fifth** | **Missing** |
| **N** | **727** | **513** | **214** | **17** | **125** | **486** | **99** | **452** | **262** | **13** | **191** | **514** | **22** | **81** | **158** | **72** | **119** | **198** | **99** |
| **Mean (SD)** |  | | | | | | | | | | | | | | | | | | |
| *Number of A&E attendances* | 1.03 (1.49) | 0.94 (1.44) | 1.25 (1.61) | 1.06 (1.34) | 1.05 (1.46) | 0.93 (1.41) | 1.51 (1.85) | 0.92 (1.44) | 1.21 (1.57) | 1.38 (1.50) | 0.92 (1.39) | 1.11 (1.55) | * | 0.95 (1.37) | 1.02 (1.40) | 1.04 (1.30) | 1.13 (1.63) | 0.79 (1.35) | 1.51 (1.85) |
| *Number of out of hours A&E attendances* | 0.46 (0.85) | 0.41 (0.77) | 0.57 (1.00) | 0.76 (1.25) | 0.50 (0.81) | 0.41 (0.81) | 0.56 (0.95) | 0.39 (0.82) | 0.56 (0.90) | * | 0.38 (0.81) | 0.50 (0.87) | * | 0.52 (1.01) | 0.47 (0.78) | 0.47 (0.87) | 0.48 (0.85) | 0.35 (0.75) | 0.56 (0.95) |
| *Number of A&E attendances lasting >12 hours* | 0.28 (0.63) | 0.25 (0.62) | 0.36 (0.65) | * | 0.35 (0.65) | 0.25 (0.59) | 0.36 (0.80) | 0.26 (0.58) | 0.32 (0.72) | * | 0.29 (0.71) | 0.29 (0.62) | * | 0.07 (0.26) | 0.34 (0.66) | 0.32 (0.60) | 0.21 (0.57) | 0.32 (0.66) | 0.36 (0.80) |
| *Number of A&E attendances via ambulance* | 0.83 (1.27) | 0.76 (1.22) | 1.00 (1.37) | 0.71 (1.16) | 0.82 (1.23) | 0.79 (1.27) | 1.09 (1.33) | 0.72 (1.22) | 1.02 (1.34) | 1.00 (1.15) | 0.76 (1.23) | 0.89 (1.30) | * | 0.85 (1.29) | 0.91 (1.26) | 0.90 (1.13) | 0.79 (1.40) | 0.63 (1.18) | 1.09 (1.33) |
| *Number of A&E attendances for falls* | 0.03 (0.20) | 0.02 (0.16) | 0.04 (0.26) | * | * | 0.02 (0.19) | 0.08 (0.31) | * | 0.06 (0.30) | * | * | 0.04 (0.22) | * | * | * | * | * | * | 0.08 (0.31) |

^1^Deprivation is based on LSOA of the care home

Table b) Ambulance activity for 5 month period from 1 June to 31 October. * where no mean reported as denominator <=5. Mean reports the mean of all those eligible e.g. can only have ED attendance via ambulance if you have had an ED attendance

| **Variable** | **Total** | **Sex** | | **Age** | | | | **Nursing vs residential** | | | **Dementia** | | | **Deprivation^1^** | | | | | |
| --- | --- | --- | --- | --- | --- | --- | --- | --- | --- | --- | --- | --- | --- | --- | --- | --- | --- | --- | --- |
|  |  | **Female** | **Male** | **<65** | **65-79** | **>=80** | **Missing** | **Nursing** | **Residential** | **Missing** | **No** | **Yes** | **Missing** | **Most deprived fifth** | **Second most deprived quintile** | **Middle fifth** | **Second least deprived ffith** | **Least deprived fifth** | **Missing** |
| **N** | **727** | **513** | **214** | **17** | **125** | **486** | **99** | **452** | **262** | **13** | **191** | **514** | **22** | **81** | **158** | **72** | **119** | **198** | **99** |
| **Mean (SD)** |  | | | | | | | | | | | | | | | | | | |
| *Number of ambulance call outs* | 0.46 (0.99) | 0.42 (0.90) | 0.54 (1.19) | * | 0.47 (1.22) | 0.41 (0.91) | 0.74 (1.09) | 0.41 (1.02) | 0.52 (0.91) | * | 0.54 (1.08) | 0.44 (0.97) | * | 0.85 (1.59) | 0.35 (0.88) | 0.38 (0.76) | 0.42 (0.81) | 0.30 (0.81) | 0.74 (1.09) |
| *Number of ambulance attendances* | 0.45 (0.97) | 0.42 (0.89) | 0.52 (1.14) | * | 0.45 (1.20) | 0.41 (0.89) | 0.71 (1.04) | 0.40 (0.99) | 0.52 (0.91) | * | 0.53 (1.08) | 0.43 (0.94) | * | 0.83 (1.52) | 0.35 (0.88) | 0.38 (0.76) | 0.42 (0.81) | 0.28 (0.79) | 0.71 (1.04) |
| *Number of out of hours ambulance attendances* | 0.21 (0.57) | 0.20 (0.50) | 0.25 (0.71) | * | 0.22 (0.58) | 0.20 (0.55) | 0.33 (0.69) | 0.18 (0.53) | 0.26 (0.63) | * | 0.23 (0.58) | 0.22 (0.58) | * | 0.38 (0.92) | 0.13 (0.41) | 0.24 (0.52) | 0.23 (0.51) | 0.14 (0.47) | 0.33 (0.69) |
| *Number of ambulance conveyances* | 0.29 (0.72) | 0.27 (0.67) | 0.35 (0.85) | * | 0.35 (0.98) | 0.26 (0.67) | 0.40 (0.65) | 0.27 (0.77) | 0.32 (0.62) | * | 0.38 (0.87) | 0.26 (0.67) | * | 0.60 (1.13) | 0.16 (0.64) | 0.28 (0.56) | 0.31 (0.69) | 0.20 (0.64) | 0.40 (0.65) |

^1^Deprivation is based on LSOA of the care home
